# Electroacupuncture prevents CUMS induced depressive-like behaviors by inhibiting microglia-mediated synaptic pruning induced by gut dysbiosis

**DOI:** 10.1186/s13020-026-01422-z

**Published:** 2026-05-26

**Authors:** Lu Zhang, Jingyi Quan, Linlin Nie, Shenglan He, Yeze Lin, Xiaoxiao Liu, Yifan Zhang, Lixing Zhuang, Chao Jia, Min Li, Qi Wang, Lining Duan

**Affiliations:** 1 The First Clinical Medical College, Guangzhou University of Chinese Medicine, Guangzhou, 510400 China; 2 Clinical Medical College of Acupuncture Moxibustion and Rehabilitation, Guangzhou University of Chinese Medicine, Guangzhou, 510006 China; 3 The First Affiliated Hospital of Guangzhou University of Chinese Medicine, Guangzhou, 510400 China; 4 Guangdong Clinical Research Academy of Chinese Medicine, Guangzhou, 510405 China; 5 Science and Technology Innovation Center, Guangzhou University of Chinese Medicine, Guangzhou, 510405 China; 6 Shenzhen Hospital (Futian) of Guangzhou University of Chinese Medicine, Shenzhen, 518034 China

**Keywords:** Electroacupuncture, Depression, Microglia, Synaptic pruning, Gut dysbiosis

## Abstract

**Rationale:**

Electroacupuncture (EA) has been widely used for depression treatment. Microbiota-gut-brain (MGB) axis plays a vital role in regulating emotional behaviors. However, the potential role of MGB axis in EA-mediated protective effects remains unclear.

**Methods:**

The protective effects of EA in chronic unpredictable mild stress (CUMS) induced mice were evaluated, and the gut microbiota and metabolic profiles were analyzed. Fecal microbiota transplantation (FMT) was utilized to explore the role of MGB axis in the protective effects of EA. Analyses related to synaptic pruning mediated by microglia were conducted to explore the molecular mechanisms.

**Results:**

In this study, EA treatment prevented depressive-like behaviors in CUMS mice. Mechanistically, EA ameliorated CUMS-induced gut microbiota dysbiosis and inflammation, and partially restored gut microbial metabolism, particularly affecting the abundance of *Alistipes* and taurine metabolism. Furthermore, EA significantly reduced systemic and hippocampal inflammation. It also attenuated aberrant synaptic pruning in the hippocampus. Moreover, FMT from CUMS mice induced depressive-like behaviors, gut inflammation and microglia-mediated aberrant synaptic pruning, whereas FMT from EA-treated donors exerted protective effects against these impairments.

**Conclusion:**

Collectively, our findings suggest that EA prevented CUMS-induced depression-like behaviors and support the involvement of the MGB axis in its protective effects.

**Supplementary Information:**

The online version contains supplementary material available at 10.1186/s13020-026-01422-z.

## Introduction

Depression is a leading cause of disability globally and contributes heavily to the worldwide disease burden [1]. Microbiota-gut-brain (MGB) axis is a bidirectional pathway which links peripheral gastrointestinal tract with central emotional processing regions of brain via neuronal, immune and hormonal signals [2]. Recent studies showed that the disruption of intestinal flora can trigger inflammation in the intestines and nervous system of patients with depression [3, 4]. Intestinal flora affect the development of depression via changing the gut endocrine functions and microbial metabolism components. Additionally, it has been reported that probiotics administration and fecal microbiota transplantation (FMT) existed potential antidepressant effects [3]. So, it is crucial to explore the role of MGB axis in depression development in depth for identifying novel therapeutic strategies.

There is mounting evidence that synaptic pruning is essential for proper neural circuit establishment, which occurs through every stage of a brain's development and maturation [5]. Early life stress could induce abnormal neurogenesis and synapse loss in the brain regions implicated in emotion regulation, and the reduced density of dendritic spines in individuals with depression has been attributed to excessive synaptic pruning [6]. Microglia normally engage in synaptic pruning to involve in the maintenance of the brain homeostasis [7]. However, recent studies revealed that gut dysbiosis could cause intestinal inflammation and neuroinflammation, which lead to depressive-like behaviors via activated microglia-mediated abnormal synaptic pruning in the hippocampus [8]. Therefore, it is important to investigate how signals from the intestinal flora affect microglia-mediated synaptic pruning.

Acupuncture has been broadly applied as an effective clinical intervention for depression treatment [9]. A randomized clinical trial involved in 270 patients with depression showed that electroacupuncture (EA) significantly improved the poor sleep quality, depression and anxiety symptoms after 8 weeks of intervention. Importantly, no serious adverse events related to EA were observed [10]. Moreover, acupuncture relieved the depressive behaviors in depression model mice and regulated 23 differential brain metabolites for its protective effect [11]. Notably, recent study found that EA significantly alleviated the gut dysbiosis, intestinal inflammation and neuroinflammation via MGB axis [12]. Nevertheless, whether the EA-induced antidepressant effects are mediated by MGB axis remains largely unknown.

In this work, we assessed the key role of MGB axis in EA-induced protective effects by employing a chronic unpredictable mild stress (CUMS) model and FMT models. We employed 16S rRNA gene microbiota sequencing and liquid chromatography-mass spectrometry (LC–MS) untargeted metabolomic analysis to explore the changes of gut microbiota and metabolic profiles. Moreover, we evaluated the alterations in gut barrier function, microglial polarization and synaptic pruning in the hippocampus. Our findings suggested that EA could prevent against CUMS-induced depressive-like behaviors by inhibiting microglia-mediated synaptic pruning induced by gut dysbiosis via MGB axis.

## Materials and methods

### Animals and the chronic unpredictable mild stress (CUMS) procedure

Male Swiss mice, aged 7 weeks and weighing 20–25 g, were sourced from the Guangdong Medical Laboratory Animal Center under SPF conditions, with ethical approval from Guangzhou University of Chinese Medicine (No. 20240715004). Depressive-like behaviors were induced via the CUMS procedure derived from previous research, which was conducted daily for 6 weeks [13]. Each day, mice received one randomly selected prolonged stressor and one acute stressor, neither stressor was repeated within any 3-day period. Prolonged stressors included 24 h periods of food deprivation, water deprivation, cage tilting (45°), damp bedding, and light–dark cycle reversal. Acute stressors comprised strobe light exposure (200 flashes/min for 2 h), forced swimming in ice water (4℃ for 5 min), noise exposure (80 dB for 2 h), horizontal shaking (220 rpm for 20 min), tail pinch (10 min), and restraint stress (4 h).

### Antibiotic treatment (ABX) experiment

The antibiotic intervention protocol was optimized based on previously established methodologies [14, 15]. To establish pseudo-germ-free (PGF) mice, recipient animals were orally gavaged once daily with a broad-spectrum antibiotic cocktail consisting of ampicillin (1 g/L), vancomycin (0.5 g/L), and imipenem (0.25 g/L) (all from Maclin, China) for 7 consecutive days prior to FMT. To further inhibit fungal overgrowth, amphotericin B (1 mg/kg) was administered during the first 3 days.

### Fecal microbiota transplantation (FMT) procedure

The FMT protocol was adapted from established methods with specific modifications [15, 16]. To ensure complete clearance of antibiotic residues from the intestinal tract, mice received a PEG-based lavage solution following ABX treatment. The solution formulation consisted of (in g/L): PEG4000 59, NaCl 1.46, KCl 0.75, Na₂SO₄ 5.68, and NaHCO₃ 1.68 (all from Maclin, China). This solution was administered via oral gavage on three occasions: twice before FMT (2 × 500 μL), with a one-hour interval, and once (1 × 500 μL) four hours prior to the procedure. Mice were fasted for 4 h before each lavage. For FMT, we homogenized 1 g of donor feces in 10 mL of sterile PBS. After thorough mixing for 5 min, the suspension was filtered through a 100 μm mesh to remove particulate matter. The resulting filtrate was immediately delivered to recipient mice by oral gavage (200 μL per mouse) within 10 min of preparation to maintain microbial viability.

### Experimental groups and treatments

The mice were randomly allocated into four distinct experimental groups: (1) The Control group, which received standard housing conditions without any interventions; (2) The CUMS group, exposed to a six-week CUMS procedure; (3) The CUMS + EA group, which underwent the six-week CUMS regimen and received EA treatment during the final three weeks. The EA intervention was performed once daily, targeting the GV20 (Baihui) and GV14 (Dazhui) acupoints with sterile, disposable acupuncture needles with specifications of 0.18 mm × 13 mm (Zhongyantaihe, China). The EA procedure was adapted from previous studies with minor modifications [13, 17]. These needles were obliquely inserted to a depth of 3 mm, and electrical stimulation (2 Hz, continuous wave) was applied for 20 min using an SDZ-III EA apparatus (Hwato, China); (4) The CUMS + FLX group was subjected to the six-week CUMS exposure and administered FLX hydrochloride (15 mg/kg/day; Macklin, China) via daily oral gavage over the last three weeks, as adapted from previous studies [13].

To further investigate the involvement of gut microbiota in depression, we designed a separate transplantation experiment. After receiving ABX pretreatment, a distinct cohort of mice was allocated into four groups for FMT: (1) The Control-FMT group, receiving daily FMT from donor mice in the Control group for a duration of six weeks; (2) The CUMS-FMT group, administered daily FMT from donors in the CUMS group for six weeks; (3) The CUMS + CUMS-FMT group, which was exposed to the six-week CUMS protocol and concurrently received daily FMT from the CUMS donor group during the final three weeks; (4) The CUMS + EA-FMT group, subjected to the six-week CUMS paradigm and given daily FMT from the CUMS + EA donor group over the last three weeks.

### Behavioral tests

The behavioral procedures used in this study were based on methods described in prior studies with minor modifications [16, 18].

Sucrose Preference Test (SPT): Animals were acclimated individually, then underwent: (1) 48 h adaptation with sucrose and water (bottles switched every 12 h); (2) 12 h water deprivation; (3) 12 h testing with pre-weighed bottles; (4) calculation of sucrose preference as sucrose intake divided by total liquid intake.

Open Field Test (OFT): All procedures were conducted under dim light with minimal noise. Mice were placed in the center of the arena for 6 min of free exploration, and behavior during the last 5 min was recorded and analyzed using VisuTrack.

Forced Swimming Test (FST) and Tail Suspension Test (TST): Behavioral despair was measured by placing mice in a water-filled cylinder (25 ± 1℃) or suspended by the tail 50 cm above a surface. Sessions lasted 6 min, with the first minute for acclimation. Immobility duration during the final 5 min was quantified automatically using VisuTrack.

### Animal tissues extraction

Before conducting behavioral tests, we transferred the mice to autoclaved clean cages lined with sterile filter paper for fecal sample collection [16]. Whenever mice defecated, we immediately collected fresh fecal pellets using sterile forceps. These samples were then transferred into enzyme-free cryogenic tubes and stored at − 80℃ for subsequent experiments and FMT procedures. After completing the final behavioral test, the mice were euthanized and collected blood samples via retro-orbital bleeding. Samples were maintained at room temperature for 2 h to facilitate complete clotting. We performed centrifugation at 3000 rpm for 15 min at 4℃ to separate serum and then stored at − 80°C. For tissue collection, we quickly dissected brain and colon tissues after euthanasia. For Golgi staining, whole brains were briefly rinsed in distilled water and immediately immersed in Golgi-Cox solution (Servicebio, China) under a fume hood. For histological analyses, brain and colon tissues were rinsed with PBS and fixed in 4% paraformaldehyde. The remaining hippocampal and colon tissues were flash-frozen and stored at − 80°C for future experiments.

### Intestinal permeability assay

Following protocols adapted from prior research [16, 19], the experimental procedure consisted of four steps: (1) Mice were fasted for 4 h and then administered FITC-dextran (600 mg/kg; FD4, 4000 MW; Beyotime, China) by oral gavage; (2) Blood samples were collected after 4 h, and separated the serum; (3) Serum samples were diluted with an equal volume of 1 × PBS and loaded into black 96-well plates at 100 μL per well; (4) Fluorescence was quantified using a microplate reader at 485/590 nm excitation/emission, with concentrations determined from a standard curve.

### Enzyme-linked immunosorbent assay (ELISA)

The concentrations of LPS, IL-6, IL-1β, and TNF-α in serum, hippocampal, and colonic samples were measured using commercial ELISA kits (all from Jianglai Bio, China) according to the manufacturer’s instructions, as described in previous studies [20]. Briefly, reagents were equilibrated to room temperature, standards were serially diluted, and tissue homogenate supernatants or serum samples were added to the wells for incubation at 37 °C for 90 min. After washing, biotinylated detection antibody and HRP-conjugated streptavidin were added sequentially, followed by substrate development in the dark and termination with stop solution. Absorbance at 450 nm was measured using a microplate reader, and sample concentrations were determined from standard curves.

### Real‑time quantitative fluorescence PCR (qRT‑PCR)

The experimental procedures for qRT-PCR were carried out as previously described, with minor modifications [21]. The extraction of total RNA from hippocampal and colon tissues was performed using TRIzol Ⅲ (Takara, Japan), followed by reverse transcription into cDNA. The experiment was conducted using a Real-Time PCR System (Applied Biosystems, USA) with 10 µL reactions that included diluted cDNA, TB Green™ Premix, gene-specific primers, and RNase-free water. The levels of gene expression were standardized and examined using the 2^–ΔΔCt^ method, with primer sequences listed in Table 1.

**Table 1:** Sequences of gene-specific primers used for RT-qPCR

Gene	Forward primer (5'–3')	Reverse primer (5'–3')
IL-4	AGTTGTCATCCTGCTCTTCTTTCTC	ATGGCGTCCCTTCTCCTGTG
IL-10	GGACAACATACTGCTAACCGACTC	TGGATCATTTCCGATAAGGCTTGG
TGF-β	AGCTGCGCTTGCAGAGATTA	AGCCCTGTATTCCGTCTCCT
IL-6	CTTCTTGGGACTGATGCTGGTGAC	AGGTCTGTTGGGAGTGGTATCCTC
IL-1β	TCGCAGCAGCACATCAACAAGAG	AGGTCCACGGGAAAGACACAGG
TNF-α	GCCTCTTCTCATTCCTGCTTGTGG	GTGGTTTGTGAGTGTGAGGGTCTG
iNOS	GCCAACATGCTACTGGAGGT	TGGAGCACAGCCACATTGAT
Arg-1	AAGACAGCAGAGGAGGTGAAGAG	TAGTCAGTCCCTGGCTTATGGTTAC
CD68	CTCTTGCTGCCTCTCATCATTGG	GCTGGTAGGTTGATTGTCGTCTG
CX3CR1	CGGTCTGGTGGGAAATCTGTTG	AGGTTCAGGAGGTAGATGTCAGTG
CX3CL1	GCCGAGTCCTGCTGTCTACC	GTAGTGGACACCTGAGGAGATGG
CXCR3	TGAGCAGCACGGACACCTTC	ACCCACTGGACAGCAGCATC
SYP	TGCCTATGTGCCGCCAGAC	CACCAGGTTCAGGAAGCCAAAC
SYN	GCTTCTTCTCGTCGCTGTCTAAC	GAGCCACCGCCCACCTG
PSD95	GTCCAGTCTGTGCGAGAGGTAG	ACGGATGAAGATGGCGATAGGG
ZO-1	AGCAGTGGAAGAAGTTACAGTTGAG	AGAAGGGCTGACGGGTAAATCC
Occludin	TGGCAATGGAATAGTCAGCAACC	TCTGGTATAGAGGCTCAGCATCAC
Claudin-1	TGGGTTTCATCCTGGCTTCTCTG	CTGAGCGGTCACGATGTTGTC
Claudin-5	ACTGCCTTCCTGGACCACAAC	CGCCAGCACAGATTCATACACC
β-actin	TATGCTCTCCCTCACGCCATCC	GTCACGCACGATTTCCCTCTCAG

### Western blotting (WB) analysis

The WB protocol used in this study was adapted from earlier published studies [21]. Hippocampal tissues were lysed and centrifuged. After that, the total protein concentration was determined by a BCA assay. Following protein separation via SDS-PAGE, the proteins were transferred onto PVDF membranes and subjected to a blocking procedure. The membranes were incubated with primary antibodies (CD68, 1:1000; PSD95, 1:1000; PSD93, 1:1000; Synaptophysin, 1:1000; all from Proteintech, China) at 4 °C for 12 h, then with the secondary antibodies (anti-mouse/rabbit, 1:3000, Affinity, China). Band visualization was performed using chemiluminescence, and quantification was carried out with ImageJ software (National Institutes of Health, Bethesda, MD, USA).

### Hematoxylin and eosin (HE) staining

The protocol for HE was performed according to established procedures described in previous studies [12, 21]. Colon tissue samples were immersion-fixed in 4% paraformaldehyde for 24 h at room temperature. Following fixation, tissues underwent sequential dehydration, were embedded in paraffin blocks, and sectioned at a thickness of 4 μm. Paraffin-embedded tissue sections first underwent deparaffinization in xylene, followed by sequential rehydration through progressively diluted ethanol solutions. To enable microscopic evaluation, these sections were then subjected to staining procedures: initial treatment with hematoxylin (Servicebio, China) for 5 min, succeeded by counterstaining in eosin solution (Servicebio, China) for 30 s. After final dehydration and xylene clearing, sections were coverslipped with neutral balsam (Beyotime, China). Whole-slide images were captured using a KFBIO pathological slide scanner system.

### Immunofluorescence

Hippocampal tissues: Immunofluorescence staining of hippocampal sections was conducted based on previously reported methods, with minor modifications [16, 22]. Following embedding in molds and preparation of 20 μm frozen sections, brain tissue samples were subjected to slide-mounted staining. For antigen retrieval, sections were immersed in EDTA buffer (pH 8.0) and subjected to microwave irradiation (5 min at 800 W followed by 10 min at 400 W), then cooled to room temperature. Sections were outlined with a hydrophobic barrier pen (Gene Tech, Germany) and blocked with 3% bovine serum albumin (BSA) for 1 h at 37°C. Sections were then incubated at 4°C for 12 h with primary antibodies (Iba1, 1:200, Wako, Japan; CD68, 1:200, Proteintech, China; PSD95, 1:200, Proteintech, China) followed by appropriate Alexa Fluor-conjugated secondary antibodies (Cell Signaling Technology, USA) for 2 h at room temperature. Nuclei were counterstained with DAPI for 10 min at room temperature, and slides were coverslipped before whole-slide imaging using a KFBIO scanner (KFBIO technology, China).

Colon tissues: The protocol applied for colonic section immunofluorescence was derived from prior published studies [16, 21]. Paraffin-embedded colon sections adhered to charged slides underwent deparaffinization in xylene and graded ethanol rehydration. Subsequent processing included antigen retrieval, blocking, and immunofluorescence staining using primary antibodies against ZO-1 and Occludin (1:200, Proteintech, China), following established protocols.

### Golgi staining and analysis

The Golgi impregnation and subsequent morphometric evaluation were performed based on a protocol adapted from prior publications [23, 24]. Tissues were incubated at 26 °C for 14 days in darkness, and the solution was replaced 48 h later and was replaced every 3 days thereafter. The fixative was then replaced with tissue processing solution (Servicebio, China), refreshed after 1 h, and further incubated at 4 °C for 3 days. After that, the brains were affixed with cyanoacrylate adhesive to a vibrating microtome stage (Leica, Germany) and sectioned coronally at 60 µm. Hippocampal sections were mounted onto glass slides, air-dried, rinsed with ultrapure water, and developed in Golgi developer (Servicebio, China) for 30 min. Finally, sections were sealed with glycerol-gelatin mounting medium (Servicebio, China) and stored at room temperature protected from light. Digital whole-slide images were obtained using a Pannoramic Scanner (3DHISTECH, Hungary), with subsequent visualization performed through CaseViewer 2.4 software (3DHISTECH, Hungary). Quantitative analysis of neuronal morphology and dendritic spine density was conducted utilizing ImageJ software (National Institutes of Health, Bethesda, MD, USA).

### 16S rRNA sequencing analysis

The protocol was carried out according to the standard protocols of Gene Denovo Biotechnology, and previously published studies [12, 14]. Using the HiPure Stool DNA Kit (Magen, China) to extract the total bacterial DNA from fecal samples. DNA quality was verified by assessing purity (NanoDrop 2000), concentration and integrity. The 16S rRNA V3-V4 region was amplified with 341F/806R primers, followed by purification with AMPure XP Beads and quantification using Qubit 3.0. Sequencing libraries were prepared with Illumina DNA Prep Kit, quality-checked, and sequenced on Illumina NovaSeq 6000 (PE250). Negative controls were included during both extraction and amplification to monitor contamination. The sequencing service was provided by Gene Denovo Biotechnology.

### LC–MS analysis of fecal

Fecal metabolite extraction and LC–MS/MS profiling were performed according to the standard protocol of Gene Denovo Biotechnology, referencing previously reported metabolomics workflows [12, 14]. Fecal samples were processed by lyophilization and mechanical homogenization. Using 70% aqueous methanol at 4 °C overnight, metabolites were extracted from 100 mg of the powder. After centrifugation, the supernatant was purified using solid-phase extraction cartridges and membrane filtration. The UPLC-MS/MS platform was utilized by Gene Denovo Biotechnology for additional LC–MS/MS analysis.

### Data analysis

All statistical analyses were conducted using GraphPad Prism 9.0 (GraphPad Software, USA). The normality of data distribution was evaluated with the Shapiro–Wilk test, while homogeneity of variances was verified using the Brown-Forsythe test. For comparisons between two experimental groups, either an unpaired two-tailed Student's t-test (for normally distributed data) or the Mann–Whitney U test (for non-normal distributions) was employed. Multiple group comparisons were performed using one-way ANOVA with Tukey's post hoc test when parametric assumptions were satisfied; otherwise, the Kruskal–Wallis test followed by Dunn's post hoc correction was applied. Correlation analyses were conducted using Spearman's rank method through OmicStudio tools. A probability value of *P* < 0.05 was considered statistically significant. All data are expressed as mean ± standard error of the mean (SEM). Additional detailed statistical data are provided in the Supplementary File 1: Table S1–S7.

## Result

### EA treatment prevented CUMS-induced depressive-like behaviors

**Fig. 1:**
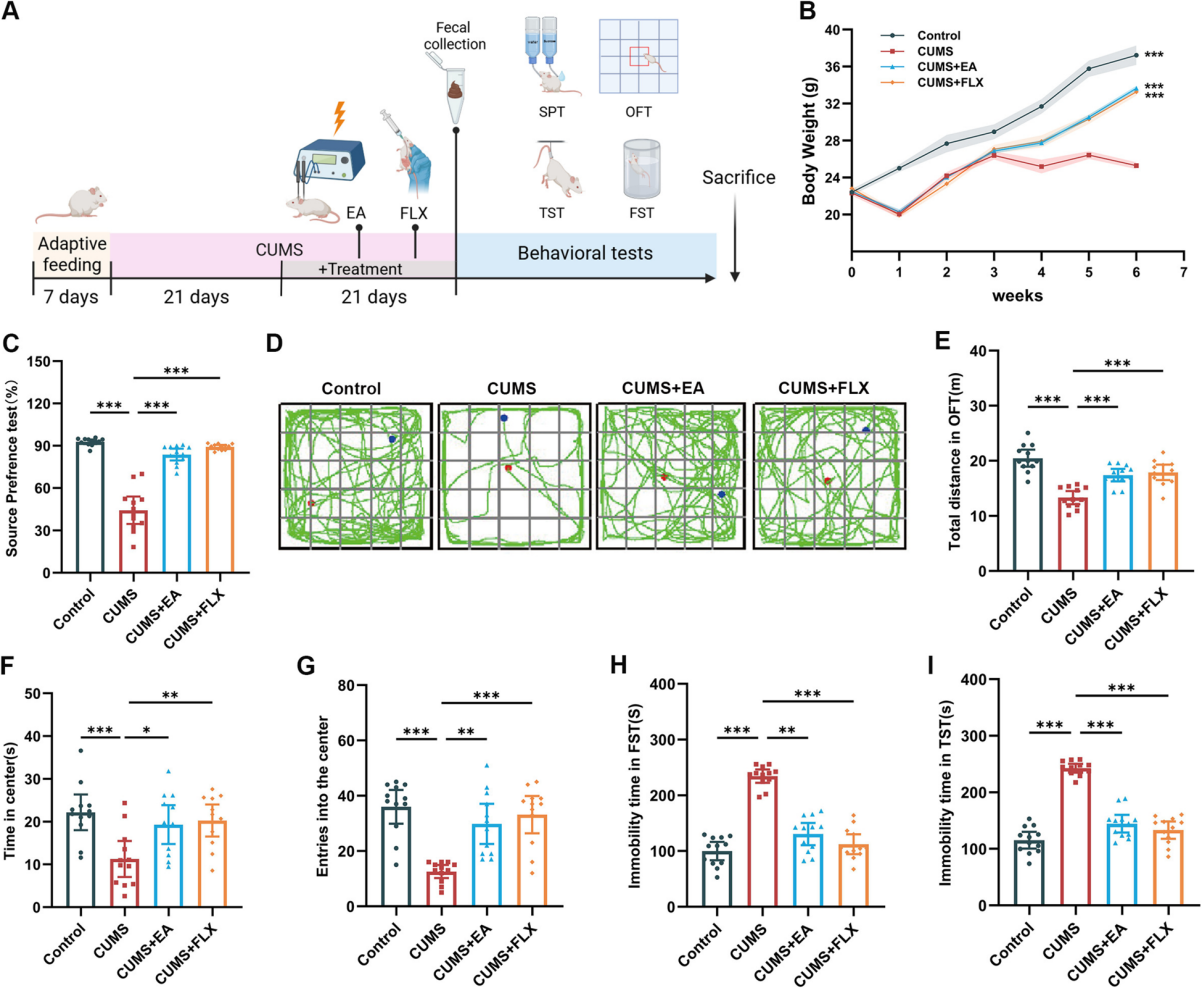
EA treatment prevented CUMS-induced depressive-like behaviors. **A** Experimental design. **B** Body weight changes of mice. **C** Sucrose preference percentage. **D** Representative movement trajectories in OFT. **E** Total distance traveled in the OFT. **F** Time spent in the center zone in the OFT. **G** Number of entries into the center zone in the OFT. **H, I** Immobility time in the FST and TST. Data are presented as mean ± SEM (n = 12). *p < 0.05, **p < 0.01, ***p < 0.001 versus the CUMS group

The research steps are shown in Fig. 1A. CUMS is an extensively-accepted model to induce depressive-like behavior in rodents. In this study, CUMS dramatically prevented the body gain in mice, while EA treatment and FLX administration significantly mitigated the inhibitory effect of CUMS on body weight increase (Fig. 1B). Moreover, CUMS remarkably reduced the sucrose preference index in SPT (Fig. 1C), reduced the total distance, time spent and entries into the center zone in OFT (Fig. 1D–G), and increased the immobility time in the FST and TST (Fig. 1H, I). Nevertheless, EA treatment effectively prevented these depressive-like behaviors and increased exploratory behaviors in the CUMS mice, with an efficacy comparable to that of FLX. Taken together, these findings suggested that EA administration could prevent CUMS-induced impairment in body weight gain and behavioral abnormalities.

### EA treatment attenuated gut microbiota dysbiosis and inflammation

**Fig. 2:**
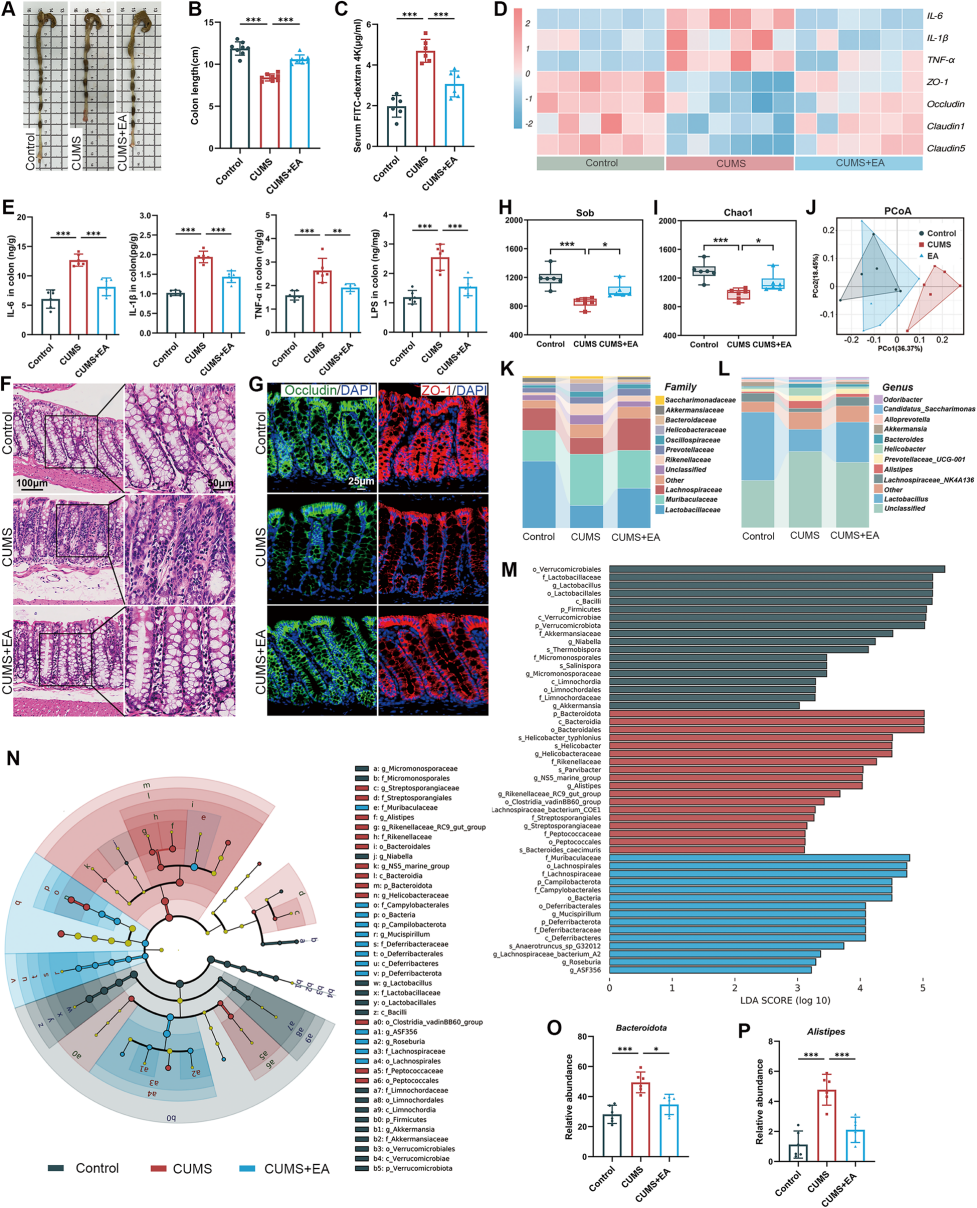
EA treatment attenuated gut microbiota dysbiosis and inflammation. **A** Representative images of colon length. **B** Quantification of colon length (n = 8). **C** Intestinal permeability assessed by serum concentration of FD4 (n = 6). **D** Heatmaps of relative mRNA expression in colon tissues. Data were z-score-normalized (n = 6). **E** Concentrations of IL-6, IL-1β, TNF-α, and LPS in the colon tissues measured by ELISA (n = 6). **F** Representative HE-stained histological sections of colon tissues (scale bar = 100 μm). **G** Representative immunofluorescence images of ZO-1 (red) and Occludin (green) in colon tissues (scale bar = 25 μm). **H, I** Analysis of alpha-diversity for the gut microbiota based on the Sob and Chao1 indices (n = 6). **J** PCoA of gut microbiota beta-diversity based on unweighted UniFrac distance (n = 6). **K, L** Bar plots of the relative abundance of the gut microbiota at the family and genus levels (n = 6). **M** Histogram of LDA scores from LEfSe identifying differentially abundant microbial taxa across the three groups (n = 6). **N** Cladogram generated by LEfSe indicating significantly enriched microbiome in each group (p: Phylum, c: Class, o: Order, f: Family, g: Genus, s: Species) (n = 6). **O, P** Relative abundance of the phylum *Bacteroidota* and the genus *Alistipes* (n = 6). Data are presented as mean ± SEM. *p < 0.05, **p < 0.01, ***p < 0.001 versus the CUMS group

Studies have shown that intestinal inflammation disrupts the integrity of the intestinal tight junction barrier, releasing LPS and inflammatory factors that can cross the blood–brain barrier (BBB), exacerbating neuroinflammation and promoting depression [25]. Therefore, we assessed the changes on gut inflammation, barrier integrity, and gut microbiota. Studies showed that EA treatment alleviated the CUMS-induced colon shortening (Fig. 2A, B) and decreased serum FD4 levels (Fig. 2C). Moreover, EA treatment inhibited the expression of IL-6, IL-1β, TNF-α and LPS at both mRNA and protein levels (Fig. 2D, E). HE staining showed that EA reduced inflammatory infiltration and epithelial injury in colon tissues (Fig. 2F). Furthermore, immunofluorescence confirmed that EA treatment restored the expression of ZO-1 and Occludin (Fig. 2G).

The integrity of the intestinal barrier is heavily dependent on the compositional state of the gut microbiota [26]. The 16S rRNA sequencing revealed that EA attenuated the CUMS-induced reduction in alpha-diversity (Sobs, Chao1; Fig. 2H, I). Beta-diversity (PCoA) showed distinct separation between the CUMS and Control groups, while EA-treated mice clustered closely with Controls (Fig. 2J). Compositional analysis revealed significant differences in gut microbiota at family and genus levels among groups (Fig. 2K, L). The linear discriminant analysis effect size pipeline (LEfSe) analysis revealed that different microbial components and biomarkers were remarkably existed from phylum to species levels between the CUMS group and Control group (Fig. 2M, N). Furthermore, CUMS administration significantly increased Bacteroidota at the phylum level and enriched *Alistipes* at the genus level, both of which were markedly reversed by EA treatment (Fig. 2O, P). Taken together, these findings indicated that EA exerted a positive effect on gut microbiota diversity and composition, and effectively attenuated the gut inflammation in CUMS mice.

### EA treatment attenuated CUMS-induced gut microbial metabolic alterations

**Fig. 3:**
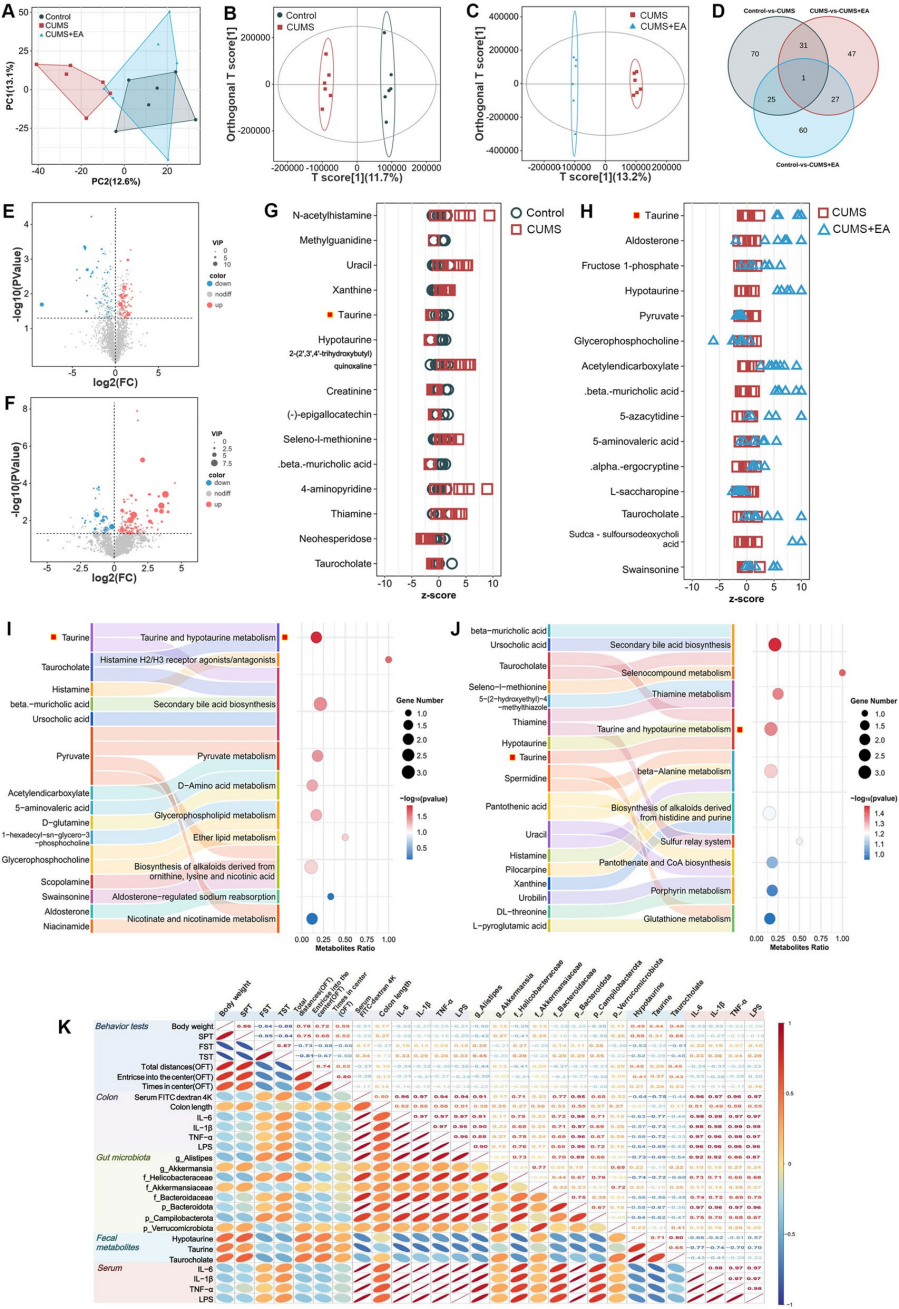
EA treatment attenuated CUMS-induced gut microbial metabolic alterations. **A** PCA of quality control (QC) samples in fecal metabolomics. **B, C** OPLS-DA score plots visualizing the discrimination of metabolic profiles between the Control and CUMS groups, and the CUMS and CUMS + EA groups. **D** Venn diagram showing the number of common and unique fecal metabolites among 3 groups. **E, F** Volcano plots of metabolites for the comparisons of Control vs. CUMS (**E**) and CUMS vs. CUMS + EA groups (**F**). Significantly upregulated (red) and downregulated (blue) metabolites were defined by VIP > 1 and *p* < 0.05. **G, H** Clustering heatmaps of the top 15 VIP-ranked differential metabolites (z-score normalized) for the comparisons of Control vs. CUMS (**G**) and CUMS vs. CUMS + EA groups (**H**). The x-axis represented the mean-centered z-scores of metabolite abundances in each group, reflecting the deviation of samples from the group mean, while the y-axis denotes the top 15 metabolites ranked by VIP score. Metabolites were defined by VIP > 1 and* p* < 0.05. **I, J** Bubble plots of KEGG pathway enrichment analysis for the comparison of Control vs. CUMS (**I**) and CUMS vs. CUMS + EA (**J**). **K** Spearman’s rank correlation analysis among behavioral tests, intestinal permeability, colon length, inflammatory mediators, significantly altered gut microbes, and differentially abundant metabolites. Correlation coefficients are visualized by bubble color (red: positive; blue: negative) and size (smaller, darker bubbles: stronger correlations). Data are presented as mean ± SEM (n = 6). *p < 0.05, **p < 0.01, ***p < 0.001 versus the CUMS group

Recent studies demonstrated that gut microbiota is able to produce a wide spectrum of biologically active metabolites, which can regulate the homeostasis of the host metabolism [27]. Therefore, we performed untargeted LC–MS metabolomic profiling of fecal samples to assess the association of EA treatment with the alterations in microbiota-derived metabolites. Principal component analysis (PCA) confirmed data reproducibility (Fig. 3A), and OPLS-DA revealed clear separations between groups (Fig. 3B, C). Venn diagram and volcano plot analysis identified significant metabolite alterations across groups (Fig. 3D–F). Z-score clustering of the top 15 VIP-ranked metabolites revealed significant distribution differences among the three groups (Fig. 3G, H). A key finding was the CUMS-induced reduction in taurine, which was partially restored by EA. Pathway analysis specifically highlighted taurine and hypotaurine metabolism as being significantly altered in both the Control vs. CUMS and CUMS vs. CUMS + EA comparisons (Fig. 3I, J). These findings suggested that EA treatment was associated with restoration of taurine metabolism in CUMS mice.

A Spearman’s rank correlation analysis integrated behavioral tests, intestinal integrity markers (colon length and permeability), inflammatory mediators (colonic and serum inflammatory cytokines, LPS), microbes and differential metabolites showed that depressive-like behaviors were positively associated with intestinal permeability, pro-inflammatory cytokines, and Gram-negative bacteria, but negatively with key metabolites. Notably, *Alistipes* showed a significant negative correlation with taurine and a strong positive correlation with LPS levels (Fig. 3K). Collectively, the above data indicated that depressive-like behaviors co-occur with disruption of intestinal homeostasis, and EA treatment was associated with reduced *Alistipes* abundance, restoration of taurine and hypotaurine metabolism, and decreased LPS concentrations.

### EA treatment attenuated microglia-mediated aberrant synaptic pruning

**Fig. 4:**
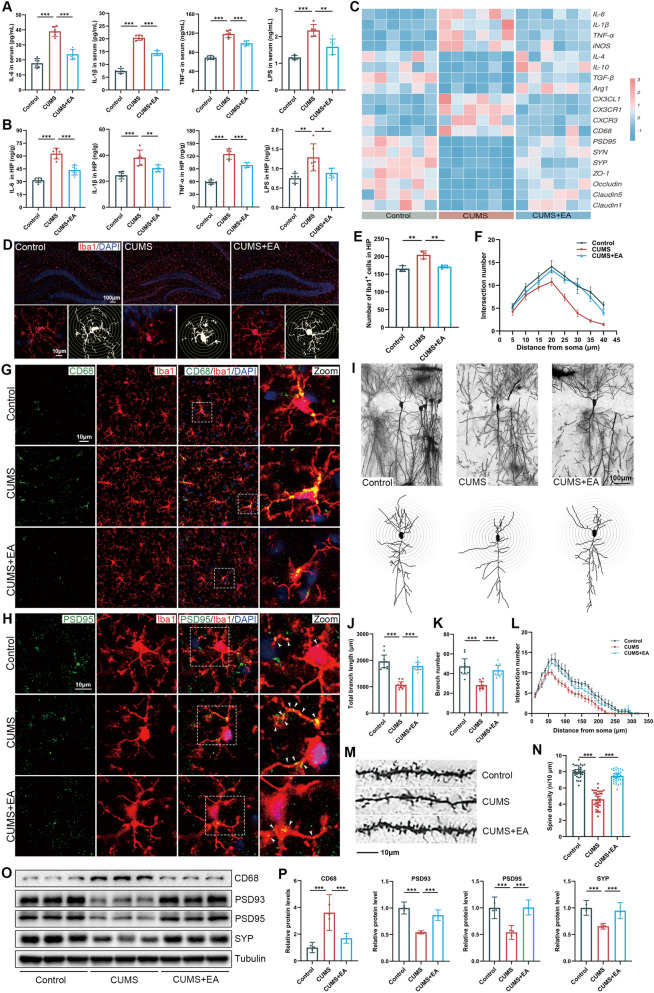
EA treatment attenuated microglia-mediated aberrant synaptic pruning. **A, B** Concentrations of IL-6, IL-1β, TNF-α, and LPS in the serum and hippocampal tissues measured by ELISA (n = 6). **C** Heatmaps showing the relative mRNA expression in the hippocampus. Data were z-score-normalized (n = 6). **D** Representative immunofluorescence images of microglial marker Iba1 (red) and Sholl analysis of microglia in the hippocampus (scale bar = 100 μm). **E** Quantification of Iba1^+^ cells (n = 3). **F** Sholl analysis of Iba1^+^ microglia morphology (n = 9 from 3 mice per group). **G** Representative immunofluorescence images of co-localization of CD68 (green) with Iba1 (red) in the hippocampus (scale bar = 100 μm). **H** Representative immunofluorescence images of co-localization of PSD95 (green) with Iba1 (red) in the hippocampus (scale bar = 100 μm). **I** Representative Golgi-stained images of hippocampal neurons (scale bar = 50 μm). **J, K** Quantification of total dendritic length and branch number of hippocampal neurons (n = 9 from 3 mice per group). **L** Number of intersections along the dendrites at all distances from the soma of hippocampal neurons (n = 9 from 3 mice per group). **M, N** Representative Golgi-stained dendritic spines of hippocampal neurons and quantification of spine density (n = 30 from 3 mice per group) (scale bar = 10 μm). **O, P** Representative images of the Western blot and relative protein expression levels of CD68, PSD93, PSD95 and SYP in the hippocampus (n = 3). Data are presented as mean ± SEM. *p < 0.05, **p < 0.01, ***p < 0.001 versus the CUMS group

Given that EA effectively inhibited gut inflammation via improving gut microbiota diversity and metabolism in CUMS mice, we then assessed the effect of EA on neuroinflammation. We found that CUMS administration increased the levels of serum and hippocampus LPS and pro-inflammatory factor IL-6, IL-1β and TNF-α in the CUMS group compared with the Control group (Fig. 4A, B). Our research showed that CUMS administration inhibited the tight junction proteins mRNA expression, whereas EA partially restored this alteration (Fig. 4C). Furthermore, immunofluorescence results based on Iba1 and Sholl analysis revealed that the number of Iba1^+^ cells were increased in the hippocampus of the CUMS group and a remarkable difference in the morphology of Iba1^+^ microglia (Fig. 4D, E), with a marked shortening and thickening of cellular processes and an increase in soma size was existed (Fig. 4D, F). Additionally, the high expression of M1 markers in the CUMS mice demonstrated that CUMS significantly promoted microglia to the pro‐inflammatory state (Fig. 4C). However, EA treatment markedly attenuated the LPS levels in the serum and hippocampus, and inhibited the microglial activation and reduced the release of inflammatory factors induced by CUMS.

Previous study demonstrated that microglia maintains synaptic structure and function [28]. Given that EA treatment inhibited excessive microglial activation, we examined whether EA treatment exhibited a protective effect against synaptic damage in CUMS mice. CD68 is an important marker reflecting the phagocytic activity of microglia [29]. We found that the protein expression of CD68 and the co-localization of CD68^+^ and Iba1^+^ signals were markedly increased in the CUMS mice. However, EA treatment attenuated the phagocytic activity of microglia in CUMS mice (Fig. 4G). Furthermore, the results of CD68, chemokine receptors CXCR3, CX3CR1 and chemokine ligand CXCL3 mRNA expression could be corroborated with the above results (Fig. 4C). The postsynaptic protein PSD95 and presynaptic marker SYP are key indicators which reflect the synaptic function [30]. In this work, the SYP and PSD95 levels were reduced in CUMS mice (Fig. 4C, O, P). Importantly, the increased co-localization of PSD95^+^ and Iba1^+^ signals in the immunofluorescence further suggested an increased interaction of activated microglia and neuronal synapse in CUMS mice. Interestingly, the co-localization of PSD95^+^ and Iba1^+^ was remarkably reduced by EA (Fig. 4H).

We further performed Golgi-Cox staining to investigate the protective potential of EA on synaptic structure (Fig. 4I–N). Sholl analysis revealed that CUMS administration reduced dendritic complexity, as indicated by decreased total branch length (Fig. 4J), reduced branch number (Fig. 4K), and altered dendritic intersection patterns (Fig. 4L). Moreover, spine density was significantly reduced (Fig. 4M, N), consistent with impaired spinogenesis and synaptic maturation. However, EA treatment partially restored the dendritic complexity in CUMS mice (Fig. 4I–N). Taken together, these results indicated that EA treatment significantly attenuated microglia-mediated aberrant synaptic pruning and protected the synaptic structure and function in the hippocampus of CUMS mice.

### CUMS-FMT induced depressive-like behaviors, gut inflammation and microglia-mediated synaptic pruning

**Fig. 5:**
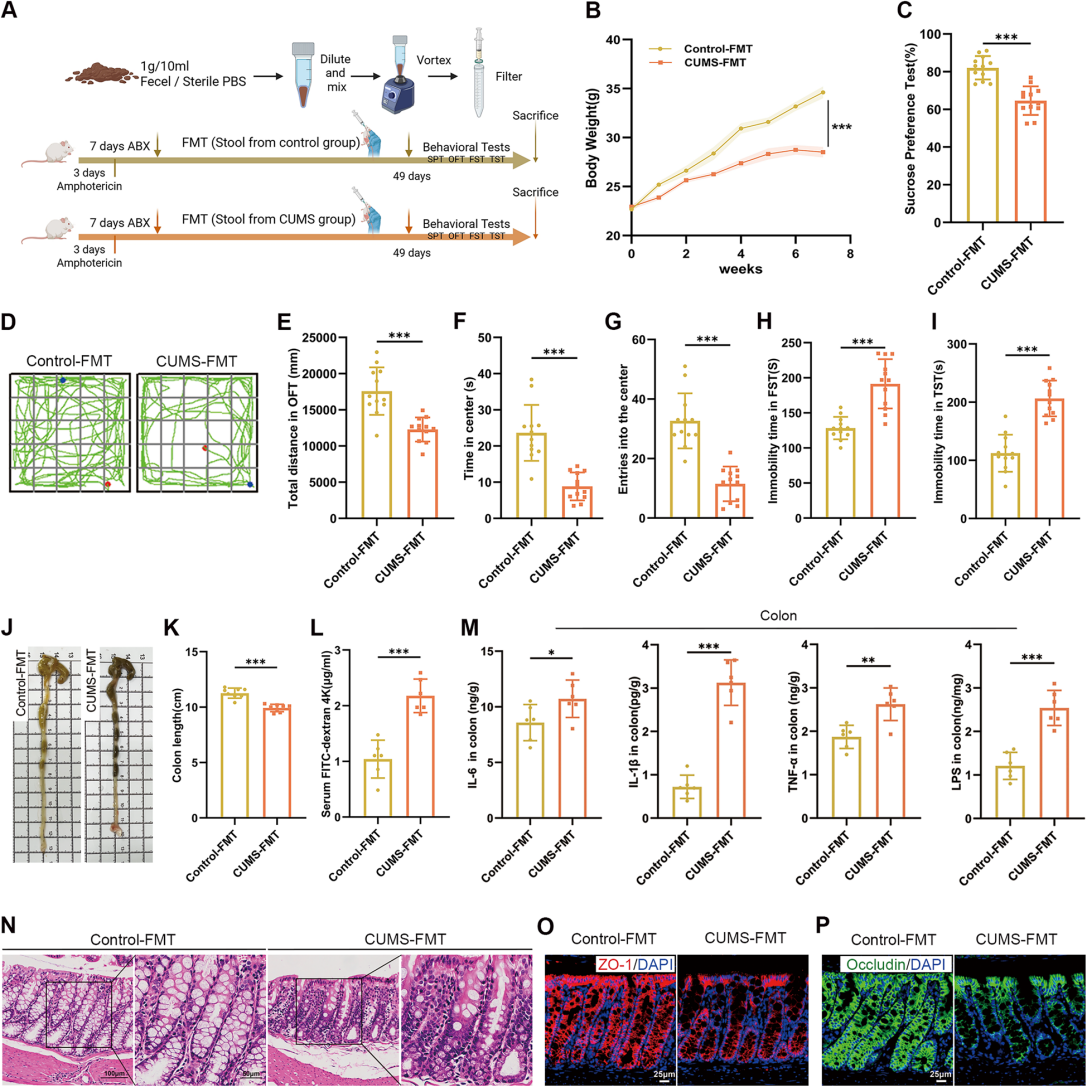
CUMS-FMT induced depressive-like behaviors and gut inflammation. **A** Experimental timeline and design of the FMT procedure. **B** Body weight changes of mice (n = 12). **C** Sucrose preference percentage (n = 12). **D** Representative movement trajectories in the OFT (n = 12). **E** Total distance traveled in the OFT (n = 12). **F** Time spent in the center zone in the OFT (n = 12). **G** Number of entries into the center zone in the OFT (n = 12). **H, I** Immobility time in the FST and TST (n = 12). **J** Representative images of colons. **K** Quantification of colon length (n = 6). **L** Intestinal permeability assessed by serum concentration of FD4 (n = 6). **M** Concentrations of IL-6, IL-1β, TNF-α, and LPS in colon tissues measured by ELISA (n = 6). **N** Representative HE-stained histological sections of colon tissues (scale bar = 100 μm). **O, P** Representative immunofluorescence images of ZO-1 (red) and Occludin (green) in the colon tissues (scale bar = 25 μm). Data are presented as mean ± SEM. *p < 0.05, **p < 0.01, ***p < 0.001 versus the Control-FMT group

**Fig. 6:**
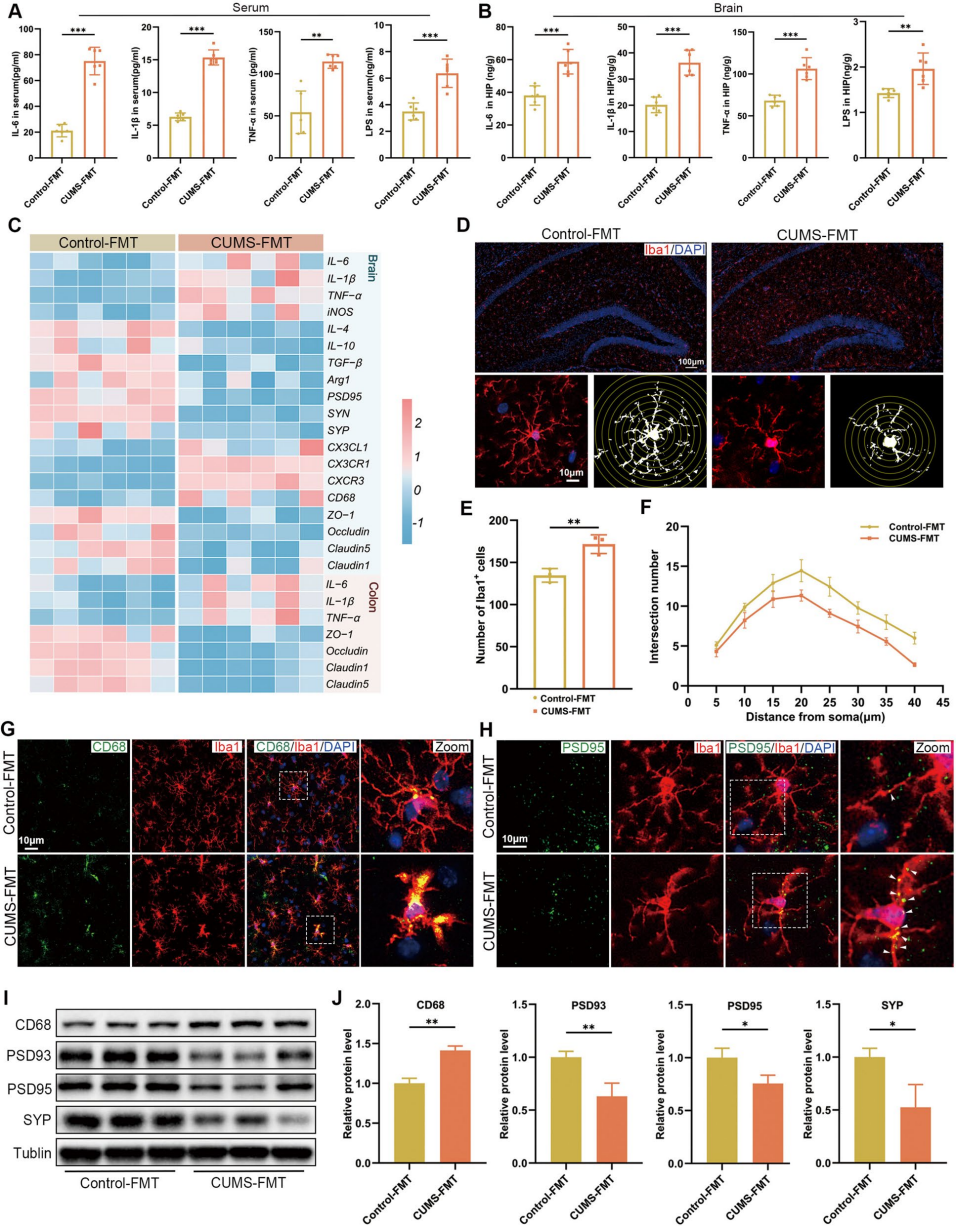
CUMS-FMT induced microglia-mediated aberrant synaptic pruning. **A, B** Concentrations of IL-6, IL-1β, TNF-α and LPS in the serum and hippocampal tissues measured by ELISA (n = 6). **C** Heatmaps showing the relative mRNA expression in the hippocampal and colon tissues. Data were z-score-normalized (n = 6). **D** Representative immunofluorescence images of microglial marker Iba1 (red) and Sholl analysis of microglia in the hippocampus (scale bar = 100 μm). **E** Quantification of Iba1^+^ cells (n = 3). **F** Sholl analysis of Iba1^+^ microglia morphology (n = 9 from 3 mice per group). **G** Representative immunofluorescence images of co-localization of CD68 (green) with Iba1 (red) in the hippocampus (scale bar = 100 μm). **H** Representative immunofluorescence images of co-localization of PSD95 (green) with Iba1 (red) in the hippocampus (scale bar = 100 μm). **I, J** Representative images of the Western blot and relative protein expression levels of CD68, PSD93, PSD95 and SYP in the hippocampus (n = 3). Data are presented as mean ± SEM. *p < 0.05, **p < 0.01, ***p < 0.001 versus the Control-FMT group

In an effort to establish a causal relationship between gut microbes and the manifestation of depressive-like behaviors, we evaluated the effect of CUMS-mediated microbiota on PGF mice via FMT derived from CUMS mice (Fig. 5A). The results revealed that PGF mice receiving the fecal microbiota from CUMS donors recapitulated the phenotype of the mice received 6-week CUMS administration (Fig. 5B–I). Next, we examined the impact of CUMS-mediated microbiota on colonic barrier function and inflammatory response. It was found that CUMS-FMT shortened colon length and increased serum FD4, indicating elevated intestinal permeability (Fig. 5J–L). Consistently, HE staining revealed notable histopathological alterations in the colon (Fig. 5N). These changes were accompanied by increased concentrations of IL-6, IL-1β, TNF-α, and LPS (Fig. 5M), together with upregulated mRNA levels of pro-inflammatory cytokines (Fig. 6C). Importantly, the levels of tight junction genes were downregulated (Fig. 6C), while ZO-1 and Occludin protein expression detected by immunofluorescence was also reduced (Fig. 5O, P).

Furthermore, it was found that PGF mice receiving gut microbiota from CUMS mice exhibited obvious concentrations of pro-inflammatory cytokines and LPS in the serum and hippocampus (Fig. 6A, B). Immunofluorescence results revealed that CUMS-FMT remarkably increased the phagocytic function of activated microglia and increased the interaction of activated microglia and neuronal synapses (Fig. 6D–H), consistent with the changes observed in RT-qPCR analysis (Fig. 6C). And the reduced protein expression levels of PSD95, PSD93 and SYP, together with increased CD68 levels, further indicated that CUMS-matched microbiota induced microglia-mediated synaptic pruning in CUMS-FMT mice (Fig. 6I, J). Collectively, these data demonstrated that FMT from CUMS-exposed donors was associated with gut barrier disruption and hippocampal microglia-associated synaptic pruning in PGF mice, in parallel with the development of depressive-like behaviors.

### EA-FMT attenuated depressive-like behaviors and gut inflammation

**Fig. 7:**
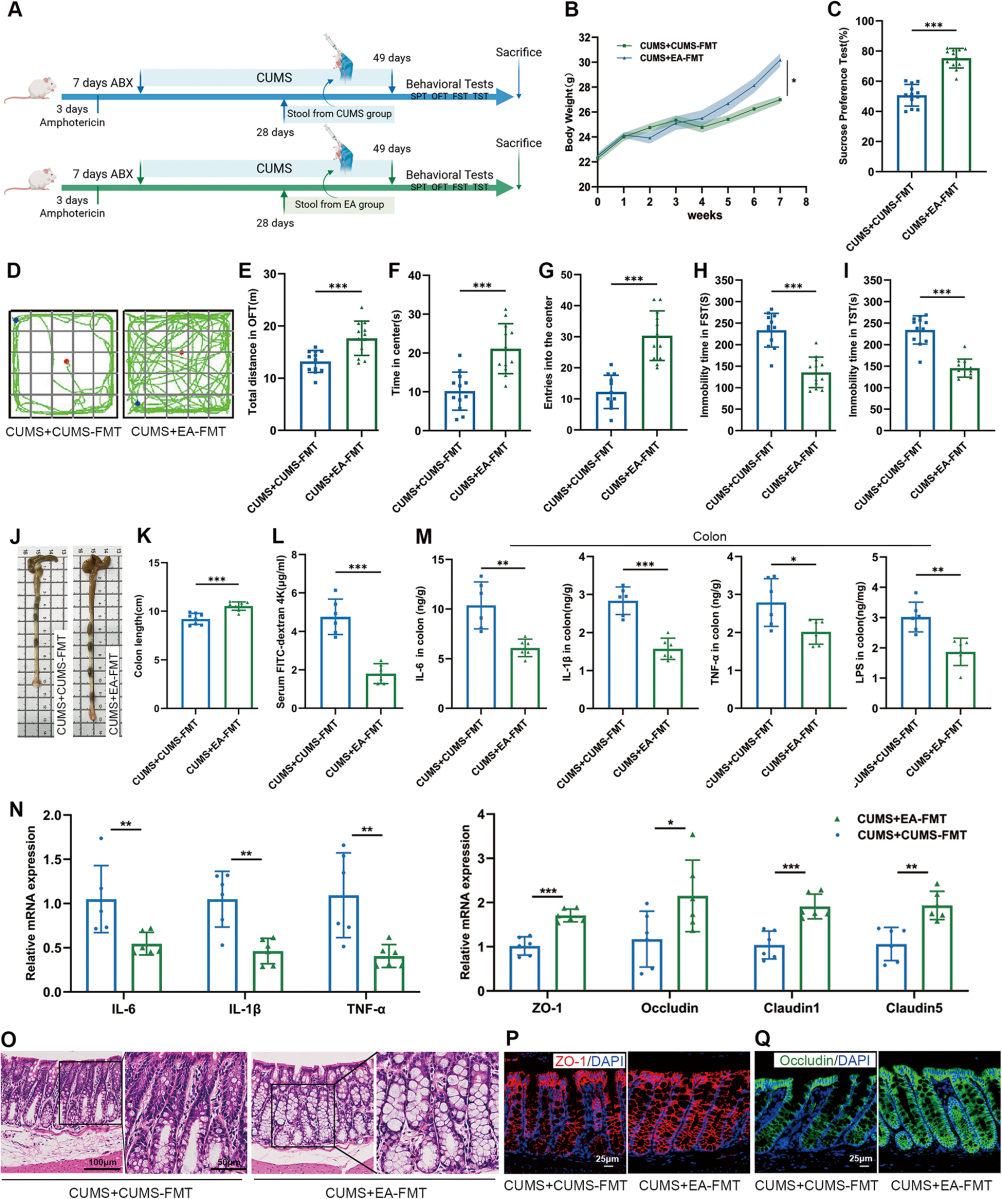
EA-FMT attenuated depressive-like behaviors and gut inflammation. **A** Experimental timeline and design of the FMT procedure. **B** Body weight changes of mice (n = 12). **C** Sucrose preference percentage (n = 12). **D** Representative movement trajectories in the OFT (n = 12). **E** Total distance traveled in the OFT (n = 12). **F** Time spent in the center zone in the OFT (n = 12). **G** Number of entries into the center zone in the OFT (n = 12). **H, I** Immobility time in the FST and TST (n = 12). **J** Representative images of the colon tissues. **K** Quantification of colon length (n = 6). **L** Intestinal permeability assessed by serum concentration of FD4 (n = 6). **M** Concentrations of IL-6, IL-1β, TNF-α, and LPS in the colon tissues measured by ELISA (n = 6). **N** Relative mRNA expression in colon tissues (n = 6). **O** Representative HE-stained histological sections of colon tissues (scale bar = 100 μm). **P, Q** Representative immunofluorescence images of ZO-1 (red) and Occludin (green) in colon tissues (scale bar = 25 μm). Data are presented as mean ± SEM. *p < 0.05, **p < 0.01, ***p < 0.001 versus the CUMS + CUMS-FMT group

FMTs were performed from CUMS or CUMS-EA mice to CUMS recipients. Before the experiment, antibiotic pretreatment was carried out to make a receptive intestinal environment for FMT (Fig. 7A). After 3 weeks of FMT, it was observed that EA-FMT significantly alleviated slow body weight gain compared with the CUMS+CUMS-FMT group (Fig. 7B). As opposed to the CUMS+CUMS-FMT group, EA-FMT effectively enhanced the sucrose preference index in SPT (Fig. 7C), increased total distance, time spent and entries into the center zone in OFT (Fig. 7D–G). Concurrently, reduced immobility durations were recorded in both the FST and TST (Fig. 7H, I). Overall, these findings demonstrated that gut microbiota from CUMS+EA mice could protect against CUMS-induced impairment in body weight gain and behavioral abnormalities.

We further assessed the effect of EA-FMT on intestinal barrier damage and inflammation in CUMS mice. Mice colonized with microbiota from the CUMS+EA group exhibited significant restoration of colon length (Fig. 7J, K) and ameliorated CUMS-induced increased intestinal permeability (Fig. 7L). Compared with the CUMS+CUMS-FMT group, EA-FMT treatment significantly downregulated the expression of pro-inflammatory cytokines at both protein and mRNA levels, and also reduced concentrations of LPS in the colon tissues (Fig. 7M, N). These anti-inflammatory effects were accompanied by a notable attenuation of elevated immune cell infiltration and epithelial damage in colon tissues (Fig. 7O). Moreover, EA-FMT treatment partially preserved intestinal barrier integrity. Evidence from mRNA measurements further confirmed the protective effect, showing increased transcript levels of essential tight junction proteins (Fig. 7N). Consistently, immunofluorescence staining of colon tissues revealed a marked increase in both the density and continuous distribution of ZO-1 and Occludin proteins in the CUMS+EA-FMT group compared to the CUMS+CUMS-FMT group, indicating improved the tight junction and structural integrity of the intestinal barrier (Fig. 7P, Q). These results demonstrated that gut microbiota derived from CUMS+EA effectively protected against intestinal barrier integrity and ameliorated colonic inflammation.

### EA-FMT attenuated microglia-mediated aberrant synaptic pruning

**Fig. 8:**
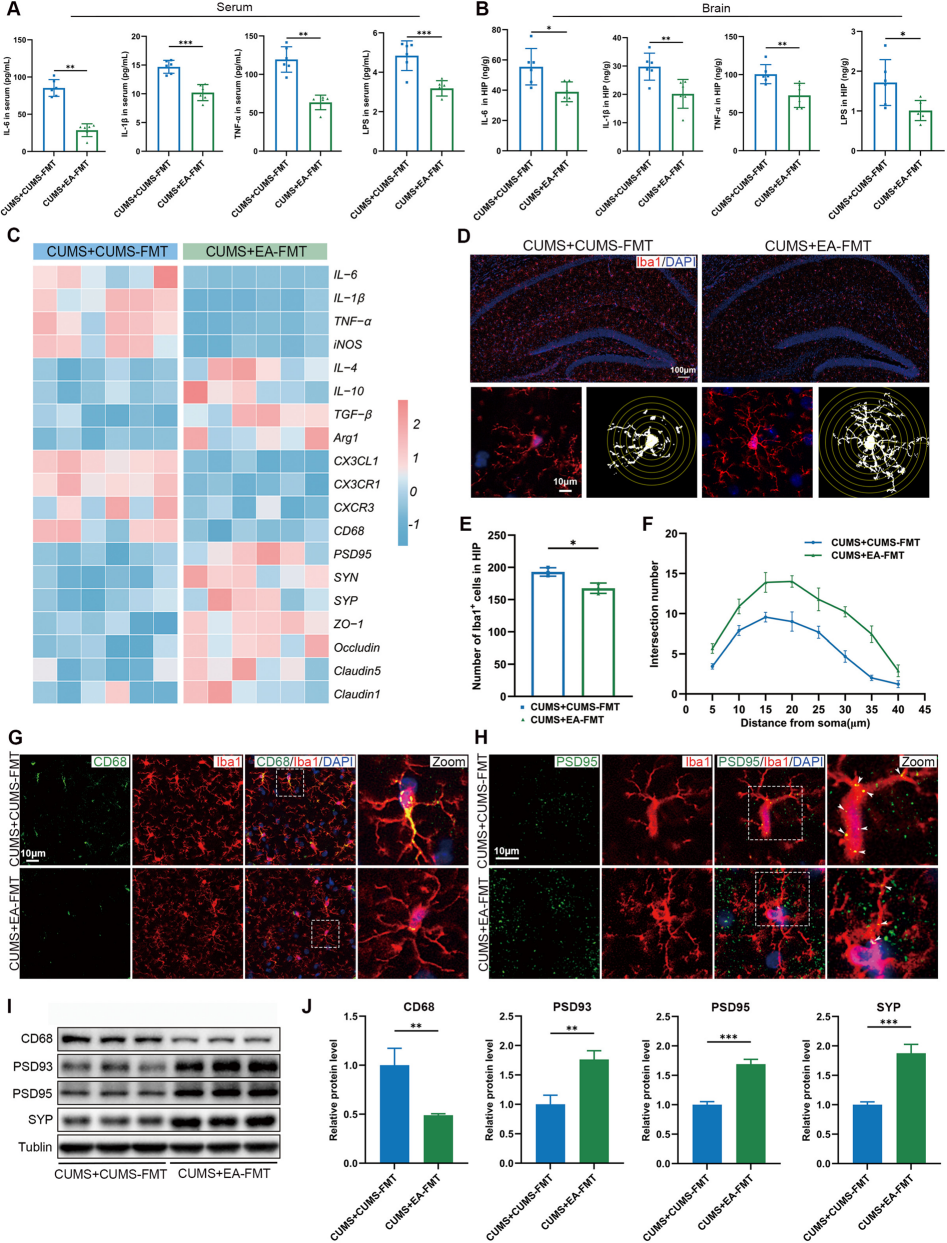
EA-FMT attenuated microglia-mediated aberrant synaptic pruning. **A, B** Concentrations of IL-6, IL-1β, TNF-α, and LPS in the serum and hippocampal tissues measured by ELISA (n = 6). **C** Heatmaps of relative mRNA expression in the hippocampus. Data were z-score normalized (n = 6). **D** Representative immunofluorescence images of microglial marker Iba1 (red) and Sholl analysis of microglia in the hippocampus (scale bar = 100 μm). **E** Quantification of Iba1^+^ cells (n = 3). **F** Sholl analysis of Iba1^+^ microglia morphology (n = 9 from 3 mice per group). **G** Representative immunofluorescence images of co-localization of CD68 (green) with Iba1 (red) in the hippocampus (scale bar = 100 μm). **H** Representative immunofluorescences images of co-localization of PSD95 (green) with Iba1 (red) in the hippocampus (scale bar = 100 μm). **I, J** Representative images of the Western blot and relative protein expression levels of CD68, PSD93, PSD95 and SYP in the hippocampus (n = 3). Data are presented as mean ± SEM. *p < 0.05, **p < 0.01, ***p < 0.001 versus the CUMS + CUMS-FMT group

To investigate whether gut microbiota transplantation from EA-treated donors influences microglial activation and synaptic pruning, we compared the outcomes between CUMS-FMT and EA-FMT treatment in a CUMS model. The findings indicated that mice subjected to CUMS and treated with EA-FMT exhibited a marked decrease in pro-inflammatory mediators within their serum and hippocampal tissues. (Fig. 8A, B), which was further confirmed by RT-qPCR analysis (Fig. 8C). We next found that EA-FMT upregulated the expression of tight junction genes (Fig. 8C). Subsequently, immunofluorescence analysis revealed that EA-FMT suppressed microglial activation in the hippocampus (Fig. 8D–F), as evidenced by reduced Iba1^+^ cell number (Fig. 8D, E) and ameliorated morphological abnormalities (Fig. 8D, F). Moreover, EA-FMT reduced the co-localization of Iba1^+^ and CD68 (Fig. 8G). Importantly, EA-FMT remarkably ameliorated the abnormal synaptic pruning by microglia in CUMS mice (Fig. 8H–J). Collectively, these results indicated that EA-FMT was associated with reduced neuroinflammatory mediators, increased tight junction gene expression, and attenuated microglia-associated synaptic pruning in CUMS mice.

Taken together, these findings show that EA-FMT prevented CUMS-induced depressive-like behaviors, gut inflammatory indices, and microglia-associated synapse pruning.

## Discussion

According to traditional Chinese medicine theory, GV20 and GV14 are commonly applied in clinical practice for brain-related disorders, while experimental studies suggest that stimulation at these acupoints may exert neuroprotective effects [11, 31]. In this work, we found that EA at GV20 and GV14 could prevent CUMS induced depressive-like behaviors. Notably, this recovery was associated with the restoration of gut microbial diversity and the amelioration of CUMS-induced disturbances in microbial metabolism and gut inflammation. In particular, EA markedly regulated the abundance of *Alistipes* as well as taurine and hypotaurine metabolism. Additionally, EA significantly reduced LPS and pro-inflammatory cytokines levels in serum. Importantly, EA reduced microglial activation and phagocytic activity, and protected against microglia-mediated synaptic pruning. Further evidence from FMT experiments suggested the crucial role of the MGB axis in these protective effects. Taken together, these findings indicate that EA may exert preventive effects against CUMS-induced depression-like behaviors, potentially through the modulation of MGB axis.

Numerous studies have established that the intestinal flora of depression patients is remarkably different from that of healthy controls [32]. Depression is frequently accompanied by chronic low-grade inflammation, which may be partly driven by gut dysbiosis, increased intestinal permeability, and immune dysregulation [33]. We found that EA intervention effectively counteracted the CUMS-induced reduction of gut microbiota alpha-diversity and beta-diversity, and ameliorated the intestinal permeability and immune microenvironment. Furthermore, LEfSe analysis showed that EA significantly mitigated CUMS-induced microbiota dysbiosis. Previous reports have indicated that *Alistipes*, a genus capable of producing harmful metabolites such as indole and hydrogen sulfide, is strongly linked to intestinal barrier disruption and neuroinflammation [34, 35]. Notably, EA significantly reduced the relative abundance of *Bacteroidota* and *Alistipes* elevated by CUMS, suggesting that EA not only restored microbial diversity at the community level, but also modulated targeted shifts in specific genera.

Moreover, it was observed that EA was associated with a marked reconfiguration of the gut metabolic profile in CUMS mice. Among the altered metabolites, taurine appeared to be a prominent metabolite, with its level significantly reduced in CUMS mice but partially restored after EA intervention. KEGG pathway enrichment analysis further identified taurine and hypotaurine metabolism as a major metabolic pathway associated with EA treatment. Several lines of evidence suggest that taurine is a neuroprotective metabolite that may participate in mood regulation through gamma-aminobutyric acid-ergic and N-methyl-D-aspartate receptor-related mechanisms, while also mitigating oxidative stress and inflammatory responses [36, 37]. Moreover, as a sulfur-containing amino acid, taurine has been demonstrated anti-inflammatory properties, which maintains mucosal immune homeostasis in the gut [14, 38]. Interestingly, correlation analysis revealed a significant inverse association between the abundance of *Alistipes* and taurine levels. These findings suggest that the anti-inflammatory effects associated with EA may be related to the reduction of *Alistipes* abundance and the improvement of taurine metabolism in the colon.

Gut microbiota dysbiosis and the consequent disruption of microbial metabolic functions represent critical contributors to intestinal homeostasis imbalance [33, 39]. Consistent with previous studies, CUMS mice exhibited marked structural and functional alterations in the intestine [40, 41]. These changes were characterized by reduced colon length, disorganized epithelial architecture, increased inflammatory cell infiltration, and significantly downregulated expression of tight junction proteins, accompanied by elevated intestinal permeability. The disruption of intestinal barrier allows the translocation of inflammatory mediators from the gut into the blood [25]. The serum levels of pro-inflammatory cytokines and LPS were substantially increased in CUMS mice, indicating the presence of systemic inflammation. Notably, EA effectively ameliorated intestinal barrier damage and inflammation, and decreased the serum LPS level. Moreover, correlation analysis showed that *Alistipes* abundance was positively associated with intestinal permeability, as well as the colonic and serum concentrations of inflammatory cytokines and LPS. In contrast, taurine levels showed significant negative correlations with these parameters. These findings further suggested that the protective effects associated with EA against CUMS-induced changes were related to a reduction in *Alistipes* abundance, decreased taurine depletion by the gut microbiota, and the attenuation of colonic inflammation and serum LPS levels.

A growing body of evidence indicates that LPS and pro-inflammatory cytokines can disrupt blood–brain barrier integrity by downregulating tight-junction-associated gene and protein expression and promoting endothelial dysfunction, which in turn contributes to facilitate neuroimmune alterations, including microglial activation [42]. The hippocampus, which plays an important role in emotion and mood related processing, appears particularly sensitive to inflammation and neuroimmune dysregulation [43, 44]. Consistent with these established mechanisms, our study found significant downregulation of tight junction mRNA in CUMS mice, along with excessive microglia activation and neuroinflammation in hippocampal tissues. Previous studies have shown that exposure to LPS and pro-inflammatory cytokines activates microglia and promotes their polarization toward a pro-inflammatory phenotype [44]. Importantly, our study showed that EA suppressed the inflammatory cytokines and LPS levels, while concurrently increasing the mRNA expression of tight junction related genes in the hippocampus. Moreover, it should be noted that EA intervention was also accompanied by reduced microglial activation and phagocytic activity. Taken together, these findings suggest that the neuroprotective changes associated with EA may involve attenuation of peripheral-to-central inflammatory signaling and reduced neuroinflammatory responses in CUMS mice.

Multiple studies have shown that hippocampal synaptic disturbances contribute to the development of depression [8]. Microglia, the resident immune cells of the central nervous system, play a pivotal role in synaptic pruning [45, 46]. Under physiological conditions, microglia help maintain the stability of neuronal circuits through selectively eliminating impaired synapses [47]. However, within the context of CUMS-induced neuroinflammation, the increased activation and phagocytic activity of microglia lead to excessive synaptic elimination [30]. Consistent with previous studies [23, 30], our immunofluorescence data showed increased co-localization of Iba1^+^ and PSD95^+^ signals in CUMS mice, suggesting enhanced microglia-synapse interactions under stress conditions. Additionally, the decreased expression of synaptic proteins SYP and PSD95, together with the results of Golgi staining, further supported the synaptic damage and excessive microglia-associated synaptic pruning in the hippocampus. In contrast, EA intervention led to reductions in microglial activation and phagocytic activity, decreased the co-localization of microglial and synaptic markers, partially restored neuronal structural complexity, and improved synaptic protein levels as well as dendritic spine density. Taken together, these findings suggest that the protective effects of EA may be closely related to the attenuation of microglia-associated excessive synaptic pruning, which contributes to preventing the development of depression.

Based on the above findings, we further confirmed the role of MGB axis in the development of depression via FMT experiments. Following the transplantation of microbiota from CUMS mice, recipient mice exhibited intestinal barrier disruption and systemic inflammatory profiles closely resembling those of donors. Moreover, the CUMS-FMT mice exhibited remarkable neuroinflammatory responses in the hippocampus, accompanied by microglial activation and enhanced phagocytic activity induced excessive synaptic pruning, ultimately leading to depressive-like behaviors. This outcome recapitulated the pathological feature observed in CUMS mice, suggesting that the disorder of MGB axis is sufficient to trigger depression. Furthermore, we investigated the role of MGB axis in the protective effect of EA. It was found that the transplantation of microbiota from EA-treated mice effectively ameliorated above pathological changes observed in CUMS recipients. In particular, EA-FMT alleviated depressive-like behaviors, attenuated gut inflammation and increased intestinal permeability, decreased serum LPS levels, and reduced microglia-associated synaptic pruning. Collectively, these results provided evidence that MGB axis could be one of the main ways through which EA treatment exerts its protective effects.

Beyond gut microbiota remodeling, the vagus nerve may contribute to the protective effects of EA by relaying intestinal signals to the brain. Increasing evidence indicates that gut microbiota-driven affective and neuroimmune alterations might be closely associated with intact vagal signaling [48, 49]. In depression-related models, subdiaphragmatic vagotomy blocks depression-like behaviors induced by fecal microbiota transplantation from depression-related donors and prevents microbiota-driven hippocampal neuroinflammation and impaired adult hippocampal neurogenesis [18]. Recent animal evidence further suggests that the regulatory effects of EA on the gut-brain axis may also depend on intact vagal signaling. In an autism spectrum disorder model, vagotomy abolished EA-associated improvements in behavior, gut microbial remodeling, and inflammatory as well as microglial abnormalities [50]. These evidence indicated that the vagus nerve may represent a mechanistic link between EA-induced improvement of the intestinal microenvironment and the attenuation of hippocampal microglia-mediated synaptic pathology in this work.

Nevertheless, there exist some limitations in the present study. First, although we compared the efficacy of EA and FLX in mice exposed to 6 weeks of CUMS, the onset profiles of their antidepressant-like effects were not evaluated. Whether these two interventions differ in the temporal dynamics of their therapeutic actions therefore remains to be determined. Second, although our findings established a key role for microglia in synaptic pruning in CUMS-exposed mice, the potential involvement of other glial populations, particularly astrocytes and oligodendrocytes, has yet to be clarified. In addition, while the MGB axis was identified as an important mediator of the beneficial effects of EA on depressive-like phenotypes, the protective actions of EA are unlikely to be confined to this pathway alone. Other molecular and cellular mechanisms should be explored in future studies. Another limitation concerns the interpretation of the behavioral findings. Decreased total distance traveled in the OFT indicates altered spontaneous locomotor activity in the CUMS mice of this study. So, increased immobility in the FST and TST should be interpreted with caution, as both tests rely on immobility-based readouts and alterations in general locomotion can act as important confounders [51, 52]. Future studies incorporating behavioral paradigms less dependent on locomotor activity would further strengthen the interpretation of the antidepressant-like effects reported here. In addition, the role of the vagus nerve in the protective effects of EA via MGB axis should be clarified in the future study.

## Conclusion

**Fig. 9:**
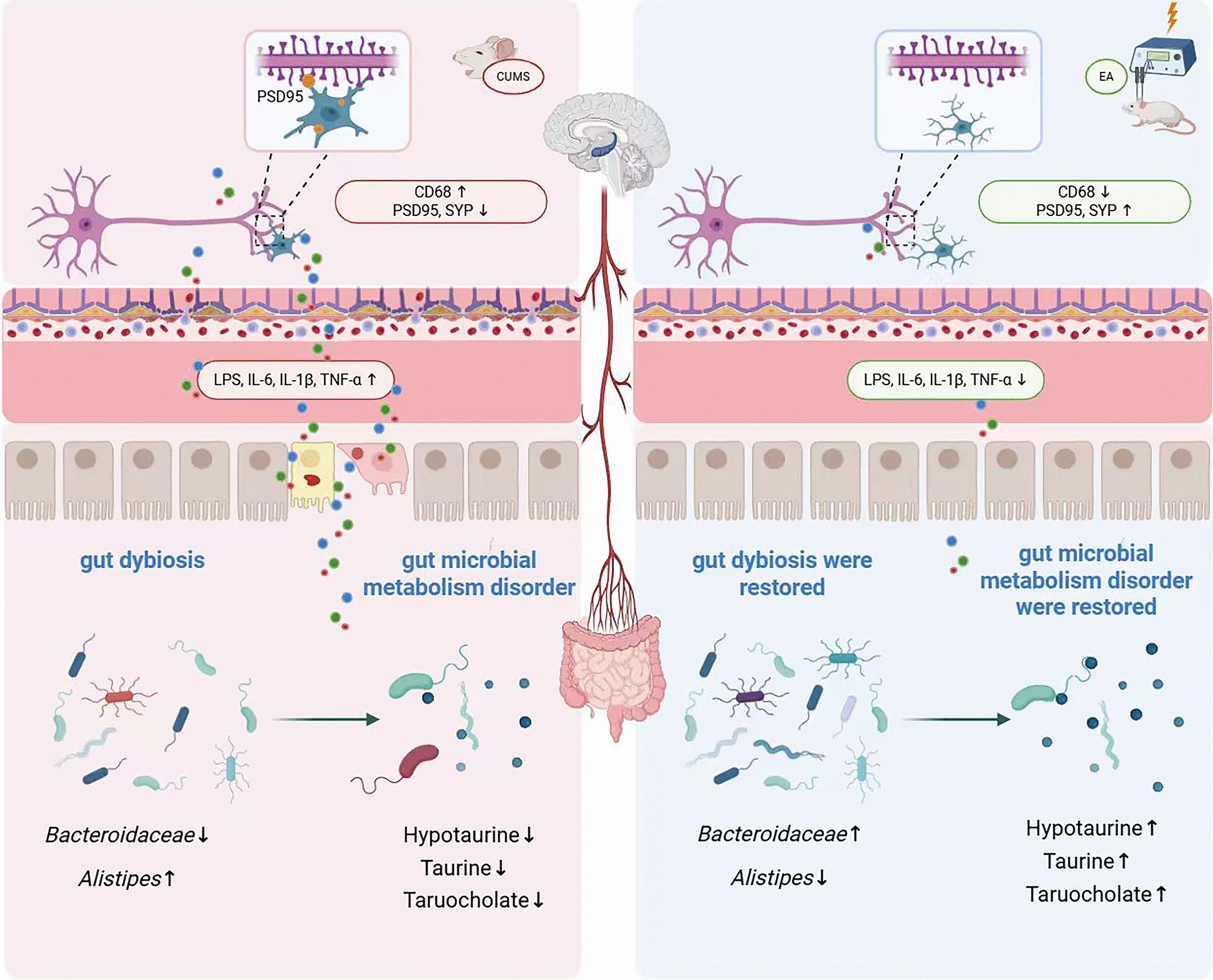
Electroacupuncture prevents CUMS induced depression-like behaviors by inhibiting microglia-mediated synaptic pruning induced by gut dysbiosis. EA treatment prevented CUMS-induced depressive-like behaviors by attenuating gut microbiota dybiosis and disturbances in microbial metabolism, which in turn reduced colonic and systemic inflammation. These peripheral improvements subsequently attenuated microglia-mediated synaptic pruning

In conclusion, our study provides compelling evidence that EA could prevent CUMS-induced depressive-like behaviors by regulating MGB axis and inhibiting microglia-mediated synaptic pruning (Fig. 9). These discoveries suggest that EA could be a viable treatment for depression and MGB axis is one of underlying mechanisms that govern its therapeutic effects. This study provides a theoretical underpinning for EA treatment clinical implementation in depression.

## Supplementary Information

**Additional file 1.**

## Data Availability

All data of this work are available from corresponding authors upon reasonable request.

## Abbreviations

ABX: *Antibiotic treatment*
BBB: *Blood-Brain Barrier*
CUMS: *Chronic Unpredictable Mild Stress*
EA: Electroacupuncture
ELISA: Enzyme-Linked Immunosorbent Assay
FD4: FITC-Dextran 4 kDa
FLX: Fluoxetine
FMT: Fecal Microbiota Transplantation
FST: Forced Swimming Test
HE: Hematoxylin and Eosin
IL-1β: Interleukin-1β
IL-6: Interleukin-6
LC-MS: *Iiquid chromatography-tandem mass spectrometry*
LEfSe: Linear Discriminant Analysis Effect Size
LPS: Lipopolysaccharide
MGB: Microbiota-Gut-Brain
OFT: *Open Field Test*
OPLS-DA: *FOrthogonal Projections to Latent Structures-Discriminant*
PCA: Principal Component Analysis
PCoA: Principal Coordinate Analysis
PGF: *Pseudo-Germ-Free*
PSD: Postsynaptic density
RT-qPCR: Reverse Transcription Quantitative Polymerase Chain
SEM: Standard error of the mean
SPF: Specific Pathogen Free
SPT: Sucrose Preference Test
SYP: *synaptophysin*
TNF-α: Tumor necrosis factor-α
TST: Tail Suspension Test
VIP: Variable Importance in Projection
WB: Western Blotting
