# Supplementary material for: Electroacupuncture prevents CUMS induced depressive-like behaviors by inhibiting microglia-mediated synaptic pruning induced by gut dysbiosis
Source: Chin Med. 2026 May 26;21:144. doi: 10.1186/s13020-026-01422-z (PMC13202871; doi:10.1186/s13020-026-01422-z)
Supplement: Supplementary file 1 — Additional file 1. [file 13020_2026_1422_MOESM1_ESM.docx]

**Supplementary File 1.** Statistical Analysis Methods and Results for the Manuscript

**Table S1.** The statistical methods and data in Figure 1.

| **Figure** | **Statistical Test** | **Main Effect / Interaction** | **Test statistic (df)** | **P value** | **Post-hoc Comparison** | **Post-hoc P value** |
| --- | --- | --- | --- | --- | --- | --- |
| Fig. 1B | Two-way ANOVA | Time ×Treatment | F (4.417, 64.785) = 38.932 | <0.0001 | CUMS vs. Control | <0.0001 |
|  |  | Time | F (1.472, 64.785) = 522.444 | <0.0001 | CUMS vs. CUMS+EA | <0.0001 |
|  |  | Treatment | F (3, 44) = 36.256 | <0.0001 | CUMS vs. CUMS+FLX | <0.0001 |
| Fig. 1C | Welch’s ANOVA | Group Effect | F (3, 22.383) = 42.533 | <0.0001 | CUMS vs. Control | <0.0001 |
|  |  |  |  |  | CUMS vs. CUMS+EA | <0.0001 |
|  |  |  |  |  | CUMS vs. CUMS+FLX | <0.0001 |
| Fig. 1E | One-way ANOVA | Group Effect | F (3, 44) = 24.2624 | <0.0001 | CUMS vs. Control | <0.0001 |
|  |  |  |  |  | CUMS vs. CUMS+EA | 0.0001 |
|  |  |  |  |  | CUMS vs. CUMS+FLX | <0.0001 |
| Fig. 1F | One-way ANOVA | Group Effect | F (3, 44) = 6.4164 | 0.0011 | CUMS vs. Control | 0.0006 |
|  |  |  |  |  | CUMS vs. CUMS+EA | 0.0123 |
|  |  |  |  |  | CUMS vs. CUMS+FLX | 0.0046 |
| Fig.1G | Kruskal-Wallis H test | Group Effect | H = 24.4637 | <0.0001 | CUMS vs. Control | <0.0001 |
|  |  |  |  |  | CUMS vs. CUMS+EA | 0.0019 |
|  |  |  |  |  | CUMS vs. CUMS+FLX | 0.0004 |
| Fig.1H | One-way ANOVA | Group Effect | F (3, 44) = 64.3328 | <0.0001 | CUMS vs. Control | <0.0001 |
|  |  |  |  |  | CUMS vs. CUMS+EA | 0.0034 |
|  |  |  |  |  | CUMS vs. CUMS+FLX | <0.0001 |
| Fig. 1I | One-way ANOVA | Group Effect | F (3, 44) = 83.4431 | <0.0001 | CUMS vs. Control | <0.0001 |
|  |  |  |  |  | CUMS vs. CUMS+EA | <0.0001 |
|  |  |  |  |  | CUMS vs. CUMS+FLX | <0.0001 |

**Table S2.** The statistical methods and data in Figure 2.

| **Figure** | **Statistical Test** | **Main Effect / Interaction** | **Test statistic (df)** | **P value** | **Post-hoc Comparison** | **Post-hoc P value** |
| --- | --- | --- | --- | --- | --- | --- |
| Fig. 2B | One-way ANOVA | Group Effect | F (2, 21) = 68.0945 | <0.0001 | CUMS vs. Control | <0.0001 |
|  |  |  |  |  | CUMS vs. CUMS+EA | <0.0001 |
| Fig. 2C  FD4 | One-way ANOVA | Group Effect | F (2, 15) = 33.7506 | <0.0001 | Control vs. CUMS | <0.0001 |
|  |  |  |  |  | CUMS vs. CUMS+EA | <0.0001 |
| Fig. 2E | One-way ANOVA | Group Effect  (IL-6) | F (2, 15) = 39.5121 | <0.0001 | Control vs. CUMS | <0.0001 |
|  |  |  |  |  | CUMS vs. CUMS+EA | <0.0001 |
|  | One-way ANOVA | Group Effect  (IL-1β) | F (2, 15) = 73.1864 | <0.0001 | Control vs. CUMS | <0.0001 |
|  |  |  |  |  | CUMS vs. CUMS+EA | <0.0001 |
|  | One-way ANOVA | Group Effect  (TNF) | F (2, 15) = 17.5220 | <0.0001 | Control vs. CUMS | <0.0001 |
|  |  |  |  |  | CUMS vs. CUMS+EA | 0.0023 |
|  | One-way ANOVA | Group Effect  (LPS) | F (2, 15) = 28.5986 | <0.0001 | Control vs. CUMS | <0.0001 |
|  |  |  |  |  | CUMS vs. CUMS+EA | 0.0001 |
| Fig. 2H | Kruskal-  Wallis H test | Group Effect  (Sobs) | H = 13.0526 | 0.0015 | Control vs. CUMS | 0.0007 |
|  |  |  |  |  | CUMS vs. CUMS+EA | 0.0463 |
| Fig. 2I | One-way ANOVA | Group Effect  (Chao 1) | F (2, 15) = 10.9259 | 0.0012 | Control vs. CUMS | 0.0003 |
|  |  |  |  |  | CUMS vs. CUMS+EA | 0.0276 |
| Fig. 2O | Kruskal-  Wallis H test | Group Effect  (Bacteroidota) | H = 12.7061 | 0.0017 | Control vs. CUMS | 0.0007 |
|  |  |  |  |  | CUMS vs. CUMS+EA | 0.0463 |
| Fig. 2P | One-way ANOVA | Group Effect  (g_Alistipes) | F (2, 15) = 27.3371 | <0.0001 | Control vs. CUMS | <0.0001 |
|  |  |  |  |  | CUMS vs. CUMS+EA | 0.0002 |

**Table S3.** The statistical methods and data in Figure 4.

| **Figure** | **Statistical Test** | **Main Effect / Interaction** | **Test statistic (df)** | **P value** | **Post-hoc Comparison** | **Post-hoc P value** |
| --- | --- | --- | --- | --- | --- | --- |
| Fig.4A | One-way ANOVA | Group Effect (IL-6) | F (2, 15) = 39.2283 | <0.0001 | Control vs. CUMS | <0.0001 |
|  |  |  |  |  | CUMS vs. CUMS+EA | 0.0002 |
|  | One-way ANOVA | Group Effect  (IL-1β) | F (2, 15) = 277.5319 | <0.0001 | Control vs. CUMS | <0.0001 |
|  |  |  |  |  | CUMS vs. CUMS+EA | <0.0001 |
|  | One-way ANOVA | Group Effect (TNF) | F (2, 15) = 122.2970 | <0.0001 | Control vs. CUMS | <0.0001 |
|  |  |  |  |  | CUMS vs. CUMS+EA | <0.0001 |
|  | Welch’s ANOVA | Group Effect (LPS) | F (2, 7.8784) = 58.8678 | <0.0001 | Control vs. CUMS | <0.0001 |
|  |  |  |  |  | CUMS vs. CUMS+EA | 0.0048 |
| Fig.4B | One-way ANOVA | Group Effect (IL-6) | F (2,15) = 61.10 | <0.0001 | Control vs. CUMS | <0.0001 |
|  |  |  |  |  | CUMS vs. CUMS+EA | <0.0001 |
|  | One-way ANOVA | Group Effect (IL-1β) | F (2,15) = 18.35 | 0.0001 | Control vs. CUMS | 0.0004 |
|  |  |  |  |  | CUMS vs. CUMS+EA | 0.0050 |
|  | One-way ANOVA | Group Effect (TNF) | F (2,15) = 107.56 | <0.0001 | Control vs. CUMS | <0.0001 |
|  |  |  |  |  | CUMS vs. CUMS+EA | 0.0005 |
|  | One-way ANOVA | Group Effect (LPS) | F (2,15) = 10.0909 | 0.0017 | Control vs. CUMS | 0.0010 |
|  |  |  |  |  | CUMS vs. CUMS+EA | 0.0102 |
| Fig.4E | One-way ANOVA | Group Effect | F (2,6) = 19.0817 | 0.0025 | Control vs. CUMS | 0.0021 |
|  |  |  |  |  | CUMS vs. CUMS+EA | 0.0052 |
| Fig. 4J | One-way ANOVA | Group Effect | F (2,24) = 38.7221 | <0.0001 | Control vs. CUMS | <0.0001 |
|  |  |  |  |  | CUMS vs. CUMS+EA | <0.0001 |
| Fig. 4K | One-way ANOVA | Group Effect | F (2,24) = 17.2583 | <0.0001 | Control vs. CUMS | <0.0001 |
|  |  |  |  |  | CUMS vs. CUMS+EA | 0.0004 |
| Fig. 4N | One-way ANOVA | Group Effect | F (2,62.4764) = 122.8980 | <0.0001 | Control vs. CUMS | <0.0001 |
|  |  |  |  |  | CUMS vs. CUMS+EA | <0.0001 |
| Fig. 4P | One-way ANOVA | Group Effect (CD68) | F (2,6) = 48.2206 | 0.0002 | Control vs. CUMS | 0.0001 |
|  |  |  |  |  | CUMS vs. CUMS+EA | 0.0004 |
|  | One-way ANOVA | Group Effect (PSD93) | F (2,6) = 129.8632 | <0.0001 | Control vs. CUMS | <0.0001 |
|  |  |  |  |  | CUMS vs. CUMS+EA | <0.0001 |
|  | One-way ANOVA | Group Effect (PSD95) | F (2,6) = 51.2599 | 0.0002 | Control vs. CUMS | 0.0002 |
|  |  |  |  |  | CUMS vs. CUMS+EA | 0.0001 |
|  | One-way ANOVA | Group Effect (SYP) | F (2,6) = 42.7008 | 0.0003 | Control vs. CUMS | 0.0004 |
|  |  |  |  |  | CUMS vs. CUMS+EA | 0.0007 |

**Table S4**. The statistical methods and data in Figure 5.

| **Figure** | **Statistical Test** | **Main Effect / Interaction** | **Test statistic (df)** | **P value** |
| --- | --- | --- | --- | --- |
| Fig. 5B | Two-way ANOVA | Time **×**Treatment | F (2,44) = 18.0784 | <0.0001 |
|  |  | Time | F (2,44) = 218.2676 | <0.0001 |
|  |  | Treatment | F (1,22) = 27.9321 | <0.0001 |
| Fig. 5C | Unpaired t test | Group Effect | t (22) = 6.1422 | <0.0001 |
| Fig. 5E | Welch’s t test | Group Effect | t (16.3) = 4.9889 | 0.0001 |
| Fig. 5F | Welch’s t test | Group Effect | t (16.1) = 5.9266 | <0.0001 |
| Fig. 5G | Unpaired t test | Group Effect | t (22) = 6.7096 | <0.0001 |
| Fig. 5H | Welch’s t test | Group Effect | t (15.3) = 5.6686 | <0.0001 |
| Fig. 5I | Unpaired t test | Group Effect | t (22) = 7.3543 | <0.0001 |
| Fig. 5K | Unpaired t test | Group Effect | t (14) = 6.7270 | <0.0001 |
| Fig. 5L | Unpaired t test | Group Effect | t (10) = 6.1415 | 0.0001 |
| Fig. 5M | Unpaired t test | Group Effect (IL-6) | t (10) = 2.2458 | 0.0485 |
|  |  | Group Effect (IL-1β) | t (10) = 9.9910 | <0.0001 |
|  |  | Group Effec (TNF-α) | t (10) = 3.9952 | 0.0025 |
|  |  | Group Effect (LPS) | t (10) = 6.3952 | <0.0001 |

**Table S5**. The statistical methods and data in Figure 6.

| **Figure** | **Statistical Test** | **Main Effect / Interaction** | **Test statistic (df)** | **P value** |
| --- | --- | --- | --- | --- |
| Fig. 6A | Unpaired t test | Group Effect (IL-6) | t (10) = 11.2620 | <0.0001 |
|  | Unpaired t test | Group Effect (IL-1β) | t (10) = 17.2358 | <0.0001 |
|  | Welch’s t test | Group Effec (TNF-α) | t (6.0365) = 5.5778 | 0.0014 |
|  | Unpaired t test | Group Effect (LPS) | t (10) = 5.6541 | 0.0002 |
| Fig. 6B | Unpaired t test | Group Effect (IL-6) | t (10) = 5.2926 | 0.0004 |
|  | Welch’s t test | Group Effect (IL-1β) | t (8.4) = 6.9882 | <0.0001 |
|  | Unpaired t test | Group Effec (TNF-α) | t (10) = 6.401 | <0.0001 |
|  | Unpaired t test | Group Effect (LPS) | t (10) = 3.648 | 0.0045 |
| Fig. 6E | Unpaired t test | Group Effect | t (4) = 4.6906 | 0.0094 |
| Fig. 6J | Unpaired t test | Group Effect (CD68) | t (4) = 8.3324 | 0.0011 |
|  | Unpaired t test | Group Effect (PSD93) | t (4) = 4.6879 | 0.0094 |
|  | Unpaired t test | Group Effect (PSD95) | t (4) = 3.5686 | 0.0234 |
|  | Unpaired t test | Group Effect (SYP) | t (4) = 3.5666 | 0.0234 |

**Table S6**. The statistical methods and data in Figure 7.

| **Figure** | **Statistical Test** | **Main Effect / Interaction** | **Test statistic (df)** | **P value** |
| --- | --- | --- | --- | --- |
| Fig. 7B | Two-way ANOVA | Time **×**Treatment | F (1.80, 39.61) = 7.426 | 0.0024 |
|  |  | Time | F (1.80, 39.61) = 136.472 | <0.0001 |
|  |  | Treatment | F (1, 22) = 1.510 | 0.2322 |
| Fig. 7C | Unpaired t test | Group Effect | t (22) = -8.785 | <0.0001 |
| Fig. 7E | Unpaired t test | Group Effect | t (22) = -3.934 | 0.0007 |
| Fig. 7F | Unpaired t test | Group Effect | t (22) = -4.680 | 0.0001 |
| Fig. 7G | Unpaired t test | Group Effect | t (22) = -6.515 | <0.0001 |
| Fig. 7H | Unpaired t test | Group Effect | t (22) = 6.406 | <0.0001 |
| Fig. 7I | Unpaired t test | Group Effect | t (22) = 7.891 | <0.0001 |
| Fig. 7K | Unpaired t test | Group Effect | t (14) = -5.178 | 0.0001 |
| Fig. 7L | Unpaired t test | Group Effect FD4 | t (10) = 6.867 | <0.0001 |
| Fig. 7M | Welch’s t test | Group Effect (IL-6) | t (6.371) = 4.165 | 0.0052 |
|  | Unpaired t test | Group Effect (IL-1β) | t (10) = 6.716 | 0.0001 |
|  | Welch’s t test | Group Effec (TNF-α) | t (7.531) = 2.667 | 0.0301 |
|  | Unpaired t test | Group Effect (LPS) | t (10) = 4.242 | 0.0017 |
| Fig. 7N | Mann-Whitney U test | Group Effect (IL-6) | U = 35.000 | 0.0043 |
|  | Unpaired t test | Group Effect (IL-1β) | t (10) = 4.154 | 0.0020 |
|  | Unpaired t test | Group Effec (TNF-α) | t (10) = 3.390 | 0.0069 |
|  | Unpaired t test | Group Effect (ZO-1) | t (10) = -6.734 | <0.0001 |
|  | Unpaired t test | Group Effect (Occludin) | t (10) = -2.337 | 0.0416 |
|  | Unpaired t test | Group Effec (Claudin1) | t (10) = -5.055 | 0.0005 |
|  | Unpaired t test | Group Effec (Claudin5) | t (10) = -4.328 | 0.0015 |

**Table S7**. The statistical methods and data in Figure 8.

| **Figure** | **Statistical Test** | **Main Effect / Interaction** | **Test statistic (df)** | **P value** |
| --- | --- | --- | --- | --- |
| Fig. 8A | Mann-Whitney U test | Group Effect (IL-6) | U = 36.000 | 0.0022 |
|  | Unpaired t test | Group Effect (IL-1β) | t (10) = 6.109 | 0.0001 |
|  | Mann-Whitney U test | Group Effec (TNF-α) | U = 36.000 | 0.0022 |
|  | Unpaired t test | Group Effect (LPS) | t (10) = 4.796 | 0.0007 |
| Fig. 8B | Unpaired t test | Group Effect (IL-6) | t (10) = 2.959 | 0.0143 |
|  |  | Group Effect (IL-1β) | t (10) = 3.371 | 0.0071 |
|  |  | Group Effec (TNF-α) | t (10) = 3.370 | 0.0071 |
|  |  | Group Effect (LPS) | t (10) = 2.746 | 0.0206 |
| Fig. 8E | Unpaired t test | Group Effect | t (4) = 4.216 | 0.0135 |
| Fig. 8J | Unpaired t test | Group Effect (CD68) | t (4) = 5.081 | 0.0071 |
|  |  | Group Effect (PSD93) | t (4) = -6.169 | 0.0035 |
|  |  | Group Effect (PSD95) | t (4) = -12.452 | 0.0002 |
|  |  | Group Effect (SYP) | t (4) = 9.551 | 0.0007 |
